# New Sesquiterpenoids From Plant-Associated *Irpex lacteus*

**DOI:** 10.3389/fchem.2022.905108

**Published:** 2022-05-17

**Authors:** Huai-Zhi Luo, Huan Jiang, Xi-Shan Huang, Ai-Qun Jia

**Affiliations:** ^1^ School of Pharmaceutical Sciences, Key Laboratory of Tropical Biological Resources of Ministry of Education, One Health Institute, Hainan University, Haikou, China; ^2^ School of Environmental and Biological Engineering, Nanjing University of Science and Technology, Nanjing, China; ^3^ State Key Laboratory for Chemistry and Molecular Engineering of Medicinal Resources, Collaborative Innovation Center for Guangxi Ethnic Medicine, School of Chemistry and Pharmaceutical Science, Guangxi Normal University, Guilin, China

**Keywords:** *Orychophragmus violaceus* (L.) O.E. Schulz, *Irpex lacteus* (Fr.) Fr, sesquiterpenoids, furan, quorum sensing

## Abstract

Bacteria produce a large number of virulence factors through the quorum sensing (QS) mechanism. Inhibiting such QS system of the pathogens without disturbing their growth is a potential strategy to control multi-drug-resistant pathogens. To accomplish this, two new tremulane-type sesquiterpenoids, irpexolaceus H (**1**) and I (**2**), along with two known furan compounds, irpexlacte B (**3**) and C (**4**), were isolated from *Orychophragmus violaceus* (L.) OE Schulz endophytic fungus *Irpex lacteus* (Fr.) Fr. Their structures were elucidated by detailed spectroscopic data (NMR, HRESIMS, IR, and UV), single-crystal X-ray diffraction, and electronic circular dichroism (ECD) analysis. Furthermore, those compounds were evaluated for anti-quorum sensing (anti-QS) activity, and compound **3** was found contributing to the potential QS inhibitory activity.

## Introduction

Bacterial quorum sensing (QS) is a cell-density-dependent communication process by which cells measure population density and trigger appropriate responses and conduct behavioral regulation, such as luminescence, motility, secretion of virulence factors, and formation of biofilms (Papenfort and Bassler, 2016). QS inhibitor (QSI) inhibits the QS system without affecting bacteria’s growth and reduces its virulence production and biofilm formation; thus, the bacteria are in a low or non-toxicity state, the growth is not inhibited, and therefore it is difficult to cause drug resistance (Jiang and Li, 2013). However, the purpose of traditional antibacterial agents is to kill or inhibit the growth of bacteria, and it is difficult to avoid bacterial resistance (Kalia, 2013). Therefore, finding new QSIs to replace traditional antibacterial agents has become a new strategy in the antibacterial field.

**FIGURE 1:**
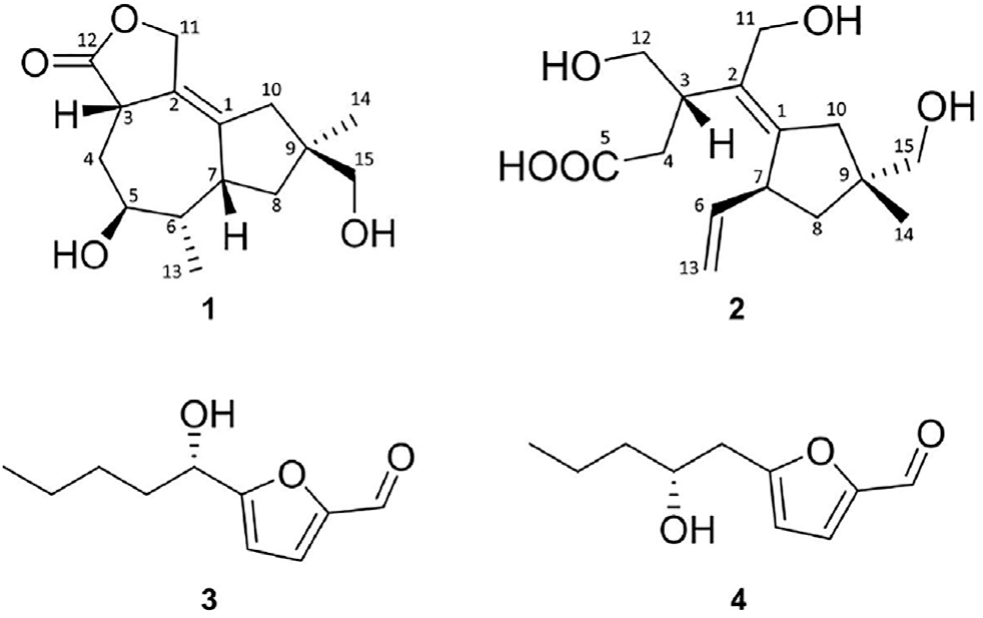
Chemical structures of **1–4**.

*Irpex lacteus* (Fr.) Fr. (Phanerochaetaceae) is a basidiomycete that usually colonizes on the deadwood white (i.e., white rot), and it is often used as a traditional Chinese medicine for the treatment of chronic glomerulonephritis. In our previous study on this subject, seven sesquiterpenoids, irpexolaceus A–G, and two new furan derivatives, irpexonjust A–B, were isolated from *I. lacteus* OV38 (Luo et al., 2022). Furthermore, two new tremulane-type sesquiterpenoids, irpexolaceus H (**1**) and I (**2**), were isolated in this study from fungi, as well as two furan compounds, such as irpexlacte B (**3**) and C (**4**), were also obtained (Figure 1). These compounds were screened for QS inhibitory activity, and compound **3** exhibited the highest QS inhibitory activity among them. The details of the isolation, structure assignment, and QS inhibitory activities of **1–4** are presented.

## Materials and Methods

### General Experimental Procedures

Nuclear magnetic resonance (NMR) was obtained on a Bruker AV-400 spectrometer (Bruker Corporation, Karlsruhe, Germany). HRMSESI data were recorded on a Q-Exactive Orbitrap MS system (Thermo Fisher Scientific, Bremen, Germany). UV data were obtained on an Evolution 220 UV-vis spectrophotometer (Thermo Fisher Scientific, Madison, United States). Infrared spectroscopy (IR) spectra were obtained on a Nicolet™ iS10 FTIR spectrometer (Thermo Fisher Scientific, Madison, United States). Optical rotations were recorded on an Autopol VI automatic polarimeter (PerkinElmer, Waltham, MA, United States). The silica gel (100–200 and 200–300 mesh, Qingdao Marine Chemical Factory, Qingdao, China) and Sephadex LH-20 column (GE Healthcare Bio-Sciences AB, Uppsala, Sweden) were used for open column chromatography (CC). Fractions were monitored by TLC (HSGF 254, Yantai Jiangyou Silica Gel Development Co., Yantai, China), and spots were visualized by heating silica gel plates after soaking in methanol supplemented with 10% H_2_SO_4_. The preparative HPLC was performed with UltiMate 300 HPLC (Thermo Fisher Scientific, Madison, United States) equipped with a YMC-Pack ODS-A column (250 × 10 mmI.D, S-5 *μ*m, 12 nm, and flow speed = 2–3 ml/min, YMC Co., Ltd., Kyoto, Japan).

### Fungal Material and Fermentation Conditions

The strain *Irpex lacteus* was isolated from the healthy flowers and stems of *Orychophragmus violaceus* (L.) OE Schulz collected from Nanjing University of Science and Technology (NJUST) (Luo et al., 2021). The fungus *I. lacteus* was fermented in 250-ml Erlenmeyer flasks containing 100 ml Fungus No. 2 medium (2% sorbitol, 2% maltose, 1% glutamine, 1% glucose, 0.3% yeast extract, 0.05% tryptophan, 0.05% KH_2_PO_4_, and 0.03% MgSO_4_, pH 6.4) at 28°C and 140 rpm for 15 d (Zhou et al., 2017).

### Extraction and Purification of Secondary Metabolites

The supernatant (total 100 L) of *I. lacteus* fermentation was extracted three times by equal ethyl acetate (EtOAc) and concentrated under reduced pressure to give a crude extract (99.49 g), which was subjected to column chromatography (CC) over a silica gel (100–200 mesh) eluted with a gradient of dichloromethane/methyl alcohol (CH_2_Cl_2_/MeOH, 100:0-0:100) to obtain a fraction G (Fr. G) and other 13 fractions (Fr. A–F and H–M). Fr. A (19.61 g) was purified by CC over a silica gel (200–300 mesh) eluted with the gradient systems of petroleum ether/EtOAc (20:1–1:1) and EtOAc/MeOH (100:1–10:1) to yield six sub-fractions Fr. A1–A6. Fr. A6 (4.79 g) was subjected to CC over a silica gel eluted with a gradient system of CH_2_Cl_2_/MeOH (80:1–0:100) to yield five sub-fractions, that is, Fr. A6.1–A6.5. Fr. A6.1 (97.4 mg) was further purified by HPLC to give **3** (40.1 mg, 45% MeOH, flow speed = 2.5 ml/min, and t_*R*_ = 31.0 min) and **4** (5.0 mg, 45% MeOH, flow speed = 2.5 ml/min, and t_*R*_ = min). Fr. G (37.30 g) was continually separated by CC over a silica gel (200–300 mesh) eluted with a gradient system of CH_2_Cl_2_/MeOH (100:0–0:100) to give nine sub-fractions Fr. G1–G9. Fr. G2 (7.76 g) was repeatedly separated using CC over a silica gel, Sephadex LH-20 (100% MeOH), and finally purified by HPLC to give **1** (30.3 mg, 50% MeOH, flow speed = 3.0 ml/min, and *t*_R_ = 14.1 min) and **2** (4.90 mg, 50% MeOH, flow speed = 3.0 ml/min, and *t*_R_ = 25.6 min).

Irpexolaceus H (**1**). white amorphous powder; [α]18 D + 17.2 (*c* 0.19, MeOH); UV (MeOH) λ_max_ (log ε) 203 (3.99) nm and 290 (2.65) nm; IR (KBr): υ_max_ 3380.60, 2932.72, 2872.93, 1752.01, and 1027.87 cm^−1^; ^1^H NMR (400 MHz in MeOD) and ^13^C NMR (100 MHz in MeOD) data are shown in Table 1; and HRESIMS *m/z* 311.1502 [M + COOH]^-^ (calcd for C_16_H_23_O_6_, 311.1500).

**TABLE 1:** ^1^H (400 MHz) and ^13^C (100 MHz) NMR spectroscopic data of **1** and **2** in CD_3_OD.

No	1		2	
	*δ*_C_, type	*δ*_H_ (*J* in Hz)	*δ*_C_, type	*δ*_H_ (*J* in Hz)
1	126.8, C	**–**	148.0, C	**–**
2	139.9, C	**–**	134.1, C	**–**
3	38.7, CH	3.62, br d (14.4)	42.1, CH	3.31, m
4	30.9, CH_2_	1.99, m and 1.84, m	40.2, CH_2_	2.27, dd (14.0 and 7.3) and 2.11, br d (15.6)
5	73.3, CH	3.96, t (5.0)	181.5, C	**–**
6	39.7, CH	1.87, m	143.6, CH	5.81, m
7	40.8, CH	3.39, m	46.3, CH	3.43, m
8	40.4, CH_2_	1.72, m; 1.40, dd (12.9 and 11.0)	43.8, CH_2_	1.93, ddd (13.2, 8.7, and 1.4) and 1.31, dd (13.2 and 7.4)
9	44.9, C	**–**	44.0, C	**–**
10	42.1, CH_2_	2.17, d (16.2) and 1.81, m	41.6, CH_2_	2.57, br d (15.6) and 2.35, dd (14.0 and 7.3)
11	70.8, CH_2_	4.82, d (13.0) and 4.73, d (13.0)	60.2, CH_2_	4.10, d (11.6) and 4.00, d (11.6)
12	182.3, C	**–**	66.4, CH_2_	3.60, dd (10.8 and 6.6); 3.50, m
13	11.8, CH_3_	0.90, d (7.0)	114.0, CH_2_	5.05, dt (17.1 and 1.48) and 4.94, br d (10.2)
14	23.9, CH_3_	1.11, s	24.5, CH_3_	1.07, s
15	68.6, CH_2_	3.21, dd (12.2 and 11.4)	69.5, CH_2_	3.26, d (11.0) and 3.21, d (11.0)

Irpexolaceus I (**2**). Yellow oil; [α]18 D + 21.6 (*c* 0.12, MeOH); UV (MeOH) λ_max_ (log ε) 205 (3.57) nm; CD (MeOH) 195 (Δε—4.72) and 215 (Δε + 8.61) nm; IR (KBr): υ_max_ 3359.87, 1564.95, 1397.66, and 1020.64 cm^−1^; ^1^H NMR (400 MHz in MeOD) and ^13^C NMR (100 MHz in MeOD) data are shown in Table 1; and HRESIMS *m/z*, 283.1551 [M-H]^-^ (calcd for C_15_H_23_O_5_ and 283.1551).

### Crystallographic Data of Compound 1

Crystal data for *Cu* ***1*** *_0m*. C_15_H_22_O_4_, *M* = 266.33, *a* = 6.6684 (4) Å, *b* = 12.1018 (5) Å, *c* = 8.4219 (4) Å, α = 90°, β = 93.164 (4)°, γ = 90°, *V* = 678.61 (6) Å^3^, *Z* = 2, *T* = 170.0 K, space group *P*2_1_2_1_2_1_, μ(Cu Kα) = 0.760 mm^−1^, *Dcalc* = 1.303 g/cm^3^, 10,065 reflections measured (10.520° ≤ 2θ ≤ 127.318°), and 2210 unique (*R*_int_ = 0.0504, *R*_sigma_ = 0.0357) which were used in all calculations. The final *R*_1_ was 0.0321 [*I* > 2σ(*I*)]. The final *wR*_2_ was 0.0752 [*I* > 2σ(*I*)]. The final *R*_1_ was 0.0346 (all data). The final *wR*_2_ was 0.0772 (all data). The goodness of fit on *F* ^2^ was 1.091. Flack parameter = −0.02 (12), which was determined using 1101 quotients [(I+)−(I−)]/[(I+)+(I−)]. CCDC: 2133065 (www.ccdc.cam.ac.uk).

### Electronic Circular Dichroism Calculation of Compound 2

The experiment was performed as previously described (Luo et al., 2022). The conformers with Boltzmann population (over 1%) were initially optimized at B3LYP/6–31+G. The ECD spectra of all conformers were conducted by time-dependent density functional theory (TD-DFT) methodology at ωB97X/def2-TZVP, CAM-B3LYP/TZVP, and M062X/def2-TZVP using IEFPCM in MeOH (Neuhaus and Loesgen, 2020). ECD spectra were generated using the program SpecDis 1.71 from dipole-length rotational strength by applying Gaussian band shapes with sigma = 0.2 eV (Bruhn et al., 2013).

### Anti-Quorum Sensing Activity

The efficacy of purified compounds on inhibiting QS mechanisms was screened using indicator organisms *Chromobacterium violaceum* CV026 and *Agrobacterium tumefaciens* A136. Overnight grown *C. violaceum* CV026 culture (1 ml) (OD_600_ ≈ 0.1) was added into 100 ml LB agar medium supplemented with kanamycin (20 μg/ml) and C6-HSL (5 μM), mixed, and poured into the plates (Kumar et al., 2021). After the medium was solidified, 2 μl of samples (**1–4**) (50 mg/ml) were dropped onto the plates and then incubated at 28°C for 24 h, recording the violacein changes in color. The absence of violacein production in CV026 represents compounds that inhibit the QS system. Furthermore, QS inhibitory activity-screened *A. tumefaciens* A136 was determined as described earlier (Zhu et al., 2018). X-gal (50 μg/ml) and C10-HSL (5 μM) were added into the LB agar medium supplemented with *A. tumefaciens* A136. Each experiment was repeated three times.

## Results and Discussion

### Structure Elucidation

**FIGURE 2:**
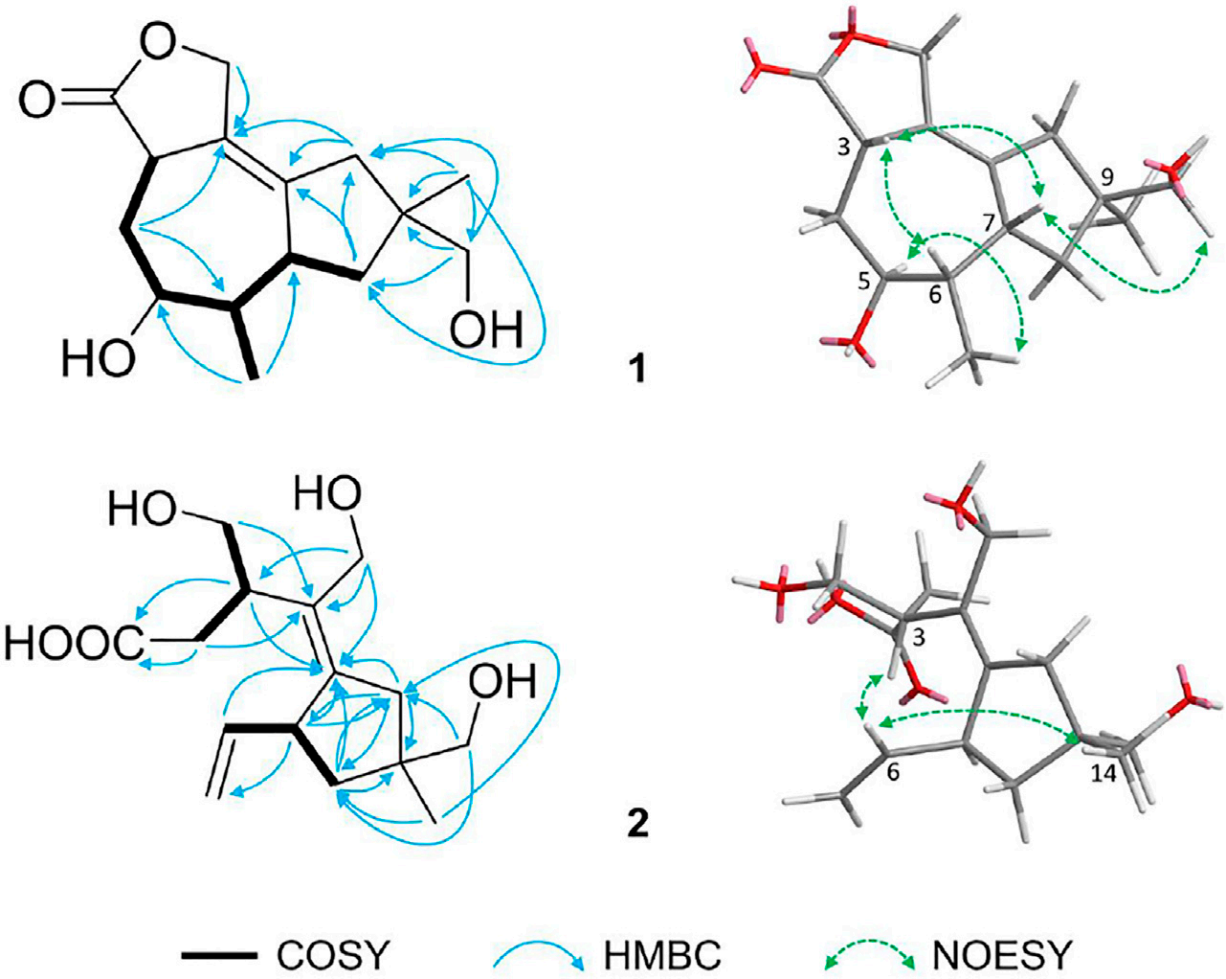
Key ^1^H–^1^H COSY (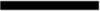), HMBC (
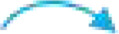
), and NOESY (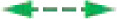) correlations of **1** and **2**.

**FIGURE 3:**
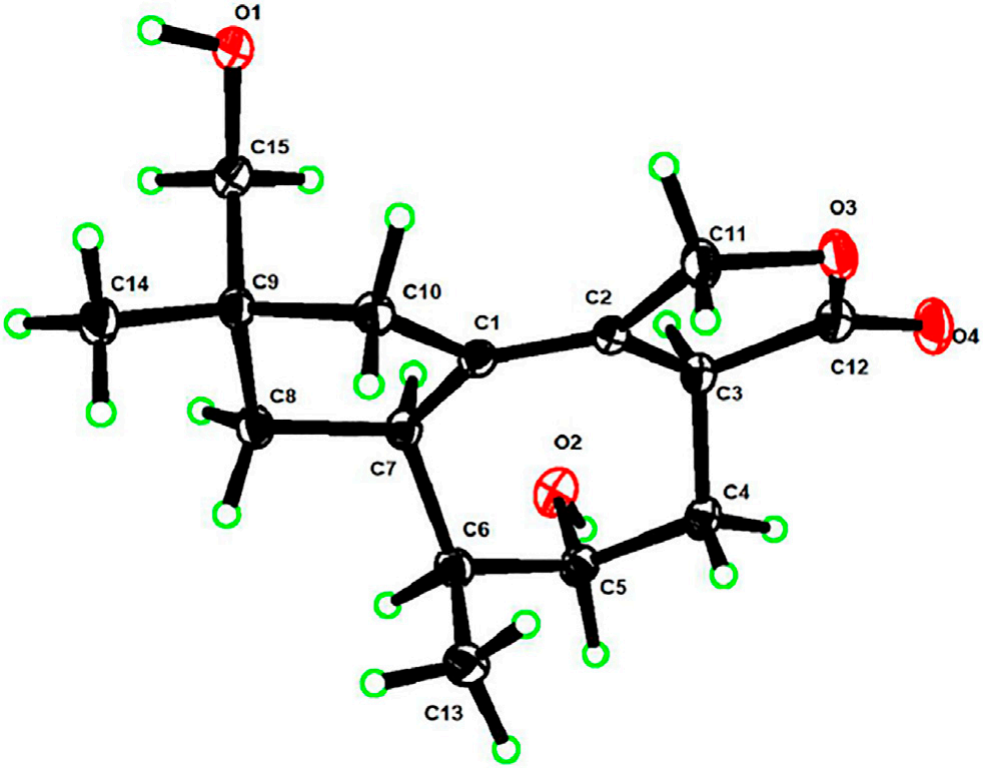
Perspective ORTEP drawing for **1**.

Irpexolaceus H (**1**)**,** a white amorphous powder, was assigned the molecular formula of C_15_H_24_O_4_ with five degrees of unsaturation based on HRESIMS data at *m/z* 311.1502 [M + COOH]^-^ (calcd for C_16_H_23_O_6_, 311.1500). The ^1^H and ^13^C NMR spectra of **1** (Table 1) revealed 15 carbon resonances, including two methyls at *δ*_C_ 11.8 and 23.9, three methylene carbons at *δ*_C_ 30.9, 40.4, and 42.1, two oxygenated methylene carbons at *δ*_C_ 70.8 and 68.6, three methine carbons at *δ*_C_ 38.7, 39.7, and 40.8, one oxygenated methine carbon at *δ*_C_ 73.3, three sp^2^ quaternary carbons at *δ*_C_ 126.8, 139.9, and 182.3, and one sp^3^ quaternary carbon at *δ*_C_ 44.9. These data demonstrated high similarity to irpexolaceus F (Luo et al., 2022), with the main difference being that the low field at *δ*_C_ 139.9 and 126.8 (**1**) shifted downfield to *δ*_C_ 165.9 and 128.5 in irpexolaceus F, indicating differences in the position of the carbon–carbon double bonds, which was confirmed by the HMBC correlations from H_2_-8 (*δ*_H_ 1.72, 1.40) and H_2_-10 (*δ*_H_ 2.17, 1.81) to C-1 (*δ*_C_ 126.8) and from H_2_-4 (*δ*_H_ 1.99, 1.84), H_2_-10 (*δ*_H_ 2.17, 1.81), and H_2_-11 (*δ*_H_ 4.82, 4.73) to C-2 (*δ*_C_ 139.9), in combination with the ^1^H–^1^H COSY correlations of H-3 (*δ*_H_ 3.62)/H_2_-4 (*δ*_H_ 1.99, 1.84)/H-5 (*δ*_H_ 3.96)/H-6 (*δ*_H_ 1.87)/H-7 (*δ*_H_ 3.39)/H_2_-8 (*δ*_H_ 1.72, 1.40) (Figure 2). The mentioned data combined with previously reported data (Luo et al., 2022) determined the planar structure of **1**. In addition, the relative configuration of **1** was determined by the NOESY correlations of H-3 (*δ*_H_ 3.62)/H-6 (*δ*_H_ 1.87)/H-7 (*δ*_H_ 3.39) and Me-13 (*δ*_H_ 0.90)/H-5 (*δ*_H_ 3.96)/Me-14 (*δ*_H_ 1.11) (Figure 2). Combined with the single-crystal X-ray diffraction analysis (Figure 3), the absolute configuration of **1** was confirmed as 3*S*,5*S*,6*S*,7*R*,9*S*.

**FIGURE 4:**
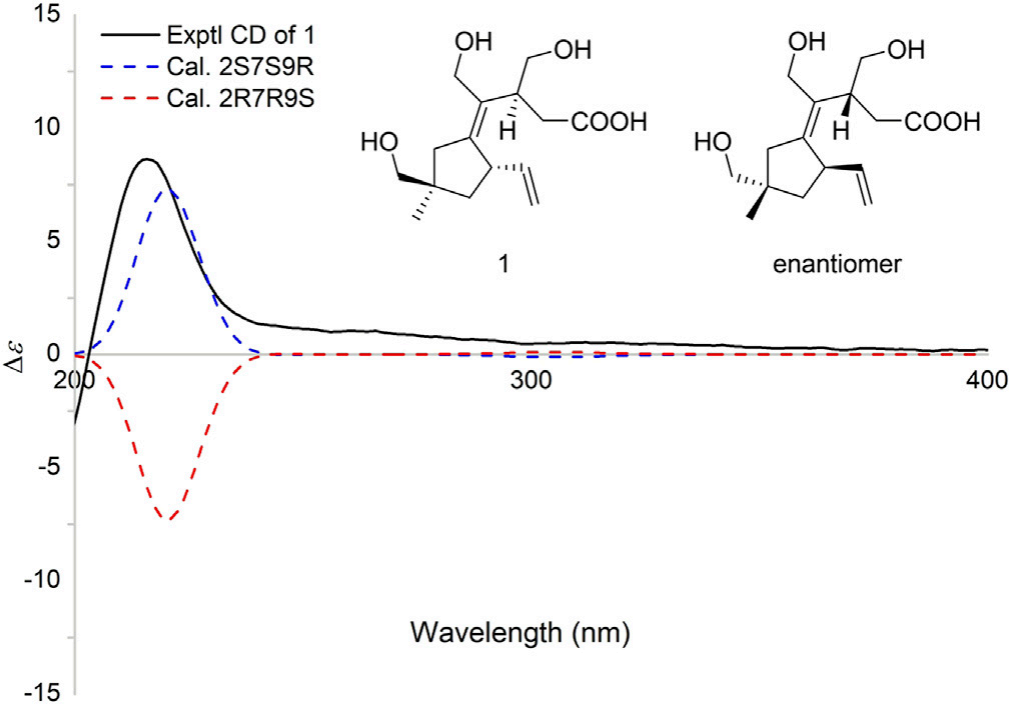
Experimental and calculated ECD spectra of **2**.

Irpexolaceus I (**2**), a yellow oil, was assigned the molecular formula of C_15_H_24_O_5_ with four degrees of unsaturation based on HRESIMS data at *m/z* 283.1551 [M-H]^-^ (calcd for C_15_H_23_O_5_, 283.1551). The ^1^H and ^13^C NMR spectra of **2** (Table 1) revealed 15 carbon resonances, including one methyl at *δ*_C_ 24.5, four methylene carbons at *δ*_C_ 40.2, 43.8, 41.6, and 114.0, three oxygenated methylene carbons at *δ*_C_ 60.2, 66.4, and 69.5, three methine carbons at *δ*_C_ 42.1, 143.6, and 46.3, three sp^2^ quaternary carbons at *δ*_C_ 148.0, 134.1, and 181.5, and one sp^3^ quaternary carbon at *δ*_C_ 44.0. There were two C=C and one carbonyl group, and the remaining one unsaturation was a ring, which was confirmed by the ^1^H-^1^H COSY correlations of H_2_-8 (*δ*_H_ 1.93, 1.31)/H-7 (*δ*_H_ 3.43)/H-6 (*δ*_H_ 5.81)/H_2_-13 (*δ*_H_ 5.05, 4.94), and the HMBC correlations from H-7 (*δ*_H_ 3.43) to C-1 (*δ*_C_ 148.0) and C-10 (*δ*_C_ 41.6), from H_2_-8 (*δ*_H_ 1.93, 1.31) to C-1 (*δ*_C_ 148.0), C-7 (*δ*_C_ 46.3), C-9 (*δ*_C_ 44.0), and C-10 (*δ*_C_ 41.6), and from H_2_-10 (*δ*_H_ 2.57, 2.35) to C-1 (*δ*_C_ 148.0), C-7 (*δ*_C_ 46.3), C-8 (*δ*_C_ 43.8), and C-9 (*δ*_C_ 44.0). Moreover, a high-field quaternary carbon, C-9 (*δ*_C_ 44.0), was linked to C-14 (*δ*_C_ 24.5) and C-15 (*δ*_C_ 69.5). Combined with another set of ^1^H–^1^H COSY correlations of H-3 (*δ*_H_ 3.31)/H_2_-4 (*δ*_H_ 2.27, 2.11)/H_2_-12 (*δ*_H_ 3.60, 3.50), the planar structure of **2** was determined as shown in Figure 1, which was highly similar to ceriponol P (Ying et al., 2014), with a difference of a hydroxyl group binding to *δ*_C_ 69.5. In addition, the relative configuration of **2** was confirmed by the NOESY correlations of H-6 (*δ*_H_ 5.81)/H-3 (*δ*_H_ 3.31)/Me-14 (*δ*_H_ 1.07) (Figure 2). The calculated and experimental ECD spectra of **2** showed excellent fit (Figure 4), indicating that the absolute configuration of **2** was 2*S*,7*S*,9*R*.

In addition to the aforementioned compounds, two furans (3 and 4) were obtained and identified as irpexlacte B and C according to the previously reported data (Luo et al., 2022).

### Proposed Biotransformation Pathway

**FIGURE 5:**
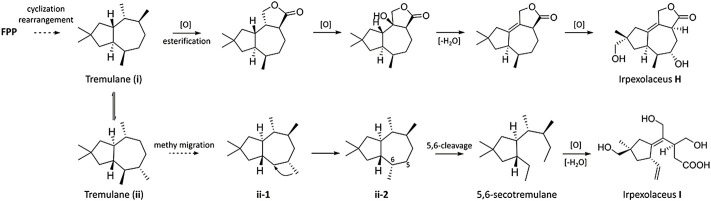
Proposed biosynthetic pathway for **1** and **2**.

Tremulane (**i**) and (**ii**) were biosynthesized based on the relative structures of **1** and **2** by the cyclization and rearrangement of farnesyl pyrophosphate (**FPP**) (Ayer and Browne, 1981; Ayer and Cruz, 1993). After a multi-step reaction such as esterification, cyclization, and dehydration in microbes, tremulane (**i**) was possibly converted to irpexlaceus H (Figure 5). Moreover, lactarane skeletons **ii-1** and **ii-2** were produced by a series of methyl migrations of tremulane (**ii**)) (Ayer and Cruz, 1993), which differed from tremulane (**i**)) in the configuration of C-1 and C-7. Lactarane skeleton **ii-2** was transformed to 5,6-secotremulane under the 5,6-cleavage (He et al., 2020) and then possibly converted to irpexlaceus I by a series of oxidation and dehydration (Figure 5).

### Biological Activity

**FIGURE 6:**
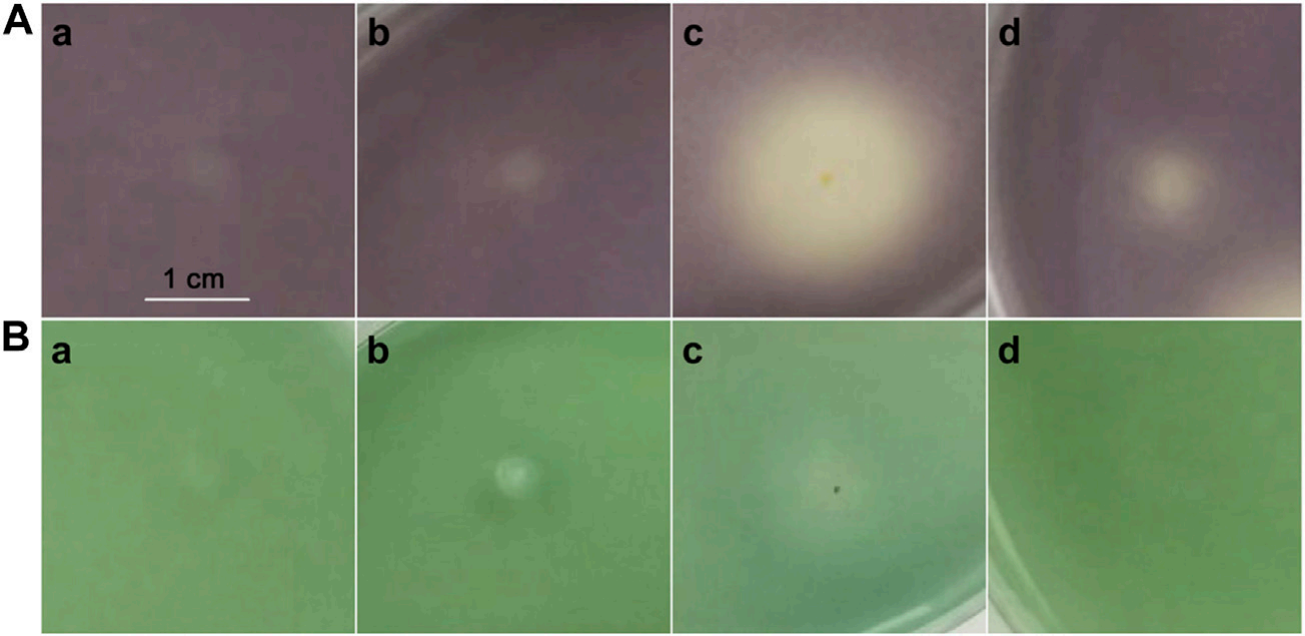
Screening of QS inhibitory activity by *C. violaceum* CV026 **(A)** and *A. tumefaciens* A136 **(B)**. Compounds 1 **(A)**, 2 **(B)**, 3 **(C),** and 4 **(D)**.

Compounds **1**–**4** were evaluated for their QS inhibitory activities at 50 mg/ml against biomarker strains *C. violaceum* CV026 (Figure 6A) and *A. tumefaciens* A136 (Figure 6B). We assessed the inhibitory effect of the compound by assaying the inhibition of C6-HSL-induced violacein production by *C. violaceum* CV026, and C10-HSL-induced *β*-galactosidase expression (blue pigment) by *A. tumefaciens* A136. Compounds **1**–**2** showed no inhibitory activities against both QS systems of biomarker strains. However, compound **3,** in which the binding position of hydroxyl was closer to the furan ring than **4**, exhibited stronger inhibition activity against the production of violacein in *C. violaceum* CV026 than that of the latter but weaker inhibitory activity against the production of blue pigment in *A. tumefaciens* A136. The results demonstrated that the binding position of hydroxyl was vital for QS inhibitory activity.

## Conclusion

Two new tremulane-type sesquiterpenoids, irpexolaceus H (**1**) and I (**2**), were isolated from the liquid fermentation of *I. lacteus*. Their structures were established based on NMR, HRESIMS, IR, single-crystal X-ray diffraction, and ECD analysis. These compounds (**1**–**4**) were evaluated for QS inhibitory activities against *C. violaceum* CV026 and *A. tumefaciens* A136 at 50 mg/ml. The results found that compound **3** exhibited a significant QS inhibitory activity against *C. violaceum* CV026, and compound **4** showed a weaker activity. In addition, compound **3** also showed a weak QS inhibitory activity against *A. tumefaciens* A136. But interestingly, the hydroxyl binding to α-C in the furan ring showed a stronger QS inhibitory activity than that of **4** (hydroxyl binding to β-C in the furan ring), which suggested that the position of hydroxyl in the furan ring was possibly vital for QS inhibitory activity against *C. violaceum* CV026.

## Data Availability

The original contributions presented in the study are publicly available. These data can be found at: https://www.ccdc.cam.ac.uk/, 2133065, 2095205, 2095201, and 2095200.

## References

[B1] Ayer W. A. Browne L. M. (1981). Terpenoid Metabolites of *Mushrooms* and Related *Basidiomycetes* . Tetrahedron 37 (12), 2197–2248. 10.1016/S0040-4020(01)97979-7

[B2] Ayer W. A. Cruz E. R. (1993). The Tremulanes, a New Group of Sesquiterpenes from the aspen Rotting Fungus *Phellinus Tremulae* . J. Org. Chem. 58 (26), 7529–7534. 10.1021/jo00078a035

[B3] Bruhn T. Schaumlöffel A. Hemberger Y. Bringmann G. (2013). SpecDis: Quantifying the Comparison of Calculated and Experimental Electronic Circular Dichroism Spectra. Chirality 25 (4), 243–249. 10.1002/chir.22138 23532998

[B4] He J. Pu C.-J. Wang M. Li Z.-H. Feng T. Zhao D.-K. (2020). Conosiligins A-D, Ring-Rearranged Tremulane Sesquiterpenoids from *Conocybe Siliginea* . J. Nat. Prod. 83 (9), 2743–2748. 10.1021/acs.jnatprod.0c00681 32816486

[B5] Jiang T. Li M. (2013). Quorum sensing Inhibitors: a Patent Review. Expert Opin. Ther. Patents 23 (7), 867–894. 10.1517/13543776.2013.779674 23506025

[B6] Kalia V. C. (2013). Quorum sensing Inhibitors: an Overview. Biotechnol. Adv. 31 (2), 224–245. 10.1016/j.biotechadv.2012.10.004 23142623

[B7] Kumar L. Brenner N. Brice J. Klein-Seetharaman J. Sarkar S. K. (2021). Cephalosporins Interfere with Quorum Sensing and Improve the Ability of *Caenorhabditis elegans* to Survive *Pseudomonas aeruginosa* Infection. Front. Microbiol. 12. 10.3389/fmicb.2021.598498 PMC7876323 33584609

[B8] Luo H.-Z. Jiang H. Sun B. Wang Z.-N. Jia A.-Q. (2022). Sesquiterpenoids and Furan Derivatives from the Orychophragmus Violaceus (L.) O.E. Schulz Endophytic Fungus Irpex Lacteus OV38. Phytochemistry 194, 112996. 10.1016/j.phytochem.2021.112996 34844037

[B9] Luo H.-Z. Zhou J.-W. Sun B. Jiang H. Tang S. Jia A.-Q. (2021). Inhibitory Effect of Norharmane on *Serratia marcescens* NJ01 Quorum Sensing-Mediated Virulence Factors and Biofilm Formation. Biofouling 37 (2), 145–160. 10.1080/08927014.2021.1874942 33682541

[B10] Neuhaus G. F. Loesgen S. (2020). Antibacterial Drimane Sesquiterpenes from *Aspergillus ustus* . J. Nat. Prod. 84 (1), 37–45. 10.1021/acs.jnatprod.0c00910 33346651

[B11] Papenfort K. Bassler B. L. (2016). Quorum sensing Signal-Response Systems in Gram-Negative Bacteria. Nat. Rev. Microbiol. 14 (9), 576–588. 10.1038/nrmicro.2016.89 27510864 PMC5056591

[B12] Ying Y.-M. Tong C.-P. Wang J.-W. Shan W.-G. Zhan Z.-J. (2014). Ceriponol P, the First Example of Monocyclic Tremulane Sesquiterpene Produced by *Ceriporia Lacerate* a Fungal Endophyte of *Huperzia Serrata* . J. Chem. Res. 38 (5), 304–305. 10.3184/174751914X13975706150476

[B13] Zhou J. Bi S. Chen H. Chen T. Yang R. Li M. (2017). Anti-biofilm and Antivirulence Activities of Metabolites from *Plectosphaerella Cucumerina* against *Pseudomonas aeruginosa* . Front. Microbiol. 8, 769. 10.3389/fmicb.2017.00769 28515715 PMC5413567

[B14] Zhu S. Wu H. Zhang C. Jie J. Liu Z. Zeng M. (2018). Spoilage of Refrigerated Litopenaeus Vannamei: Eavesdropping on Acinetobacter Acyl-Homoserine Lactones Promotes the Spoilage Potential of *Shewanella Baltica* . J. Food Sci. Technol. 55 (5), 1903–1912. 10.1007/s13197-018-3108-z 29666543 PMC5897314

