# Corrigendum: New Sesquiterpenoids From Plant-Associated *Irpex lacteus*

**DOI:** 10.3389/fchem.2022.946835

**Published:** 2022-06-24

**Authors:** Huai-Zhi Luo, Huan Jiang, Xi-Shan Huang, Ai-Qun Jia

**Affiliations:** ^1^ School of Pharmaceutical Sciences, Key Laboratory of Tropical Biological Resources of Ministry of Education, One Health Institute, Hainan University, Haikou, China; ^2^ School of Environmental and Biological Engineering, Nanjing University of Science and Technology, Nanjing, China; ^3^ State Key Laboratory for Chemistry and Molecular Engineering of Medicinal Resources, Collaborative Innovation Center for Guangxi Ethnic Medicine, School of Chemistry and Pharmaceutical Science, Guangxi Normal University, Guilin, China

**Keywords:** *Orychophragmus violaceus* (L.) O.E. Schulz, *Irpex lacteus* (Fr.) Fr, sesquiterpenoids, furan, quorum sensing

In the original article, there were some mistakes in the captions for Figures 1–3 as published. The captions of these figures were inconsistent with the figures, and the order of the figures do not accord with the description in the text. The correct figures and their captions appear below.

**FIGURE 1:**
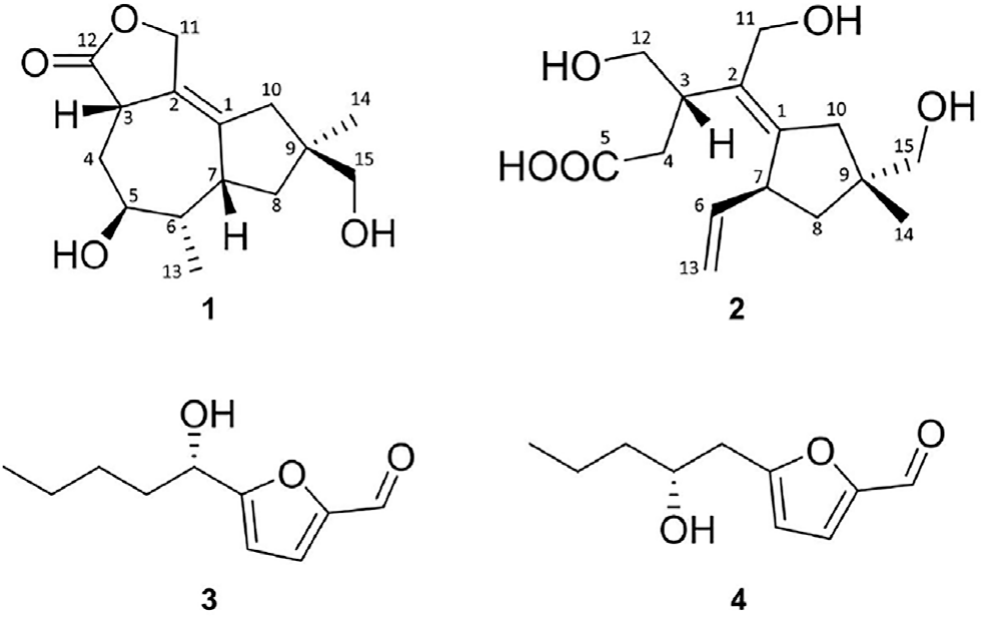
Chemical structures of **1**–**4**.

There were also errors in some of the in-text citations. In the **Introduction**, second paragraph, page 1, “Table 1” is corrected to “Figure 1” as follows: “as well as two furan compounds, such as irpexlacte B (**3**) and C (**4**), were also obtained (Figure 1).”

In **Results and Discussions**, “Structure Elucidation,” first paragraph, page 3, “Figure 1” is corrected to “Figure 2” as follows: “in combination with the ^1^H-^1^H COSY correlations of H-3 (*δ*_H_ 3.62)/H_2_-4 (*δ*_H_ 1.99, 1.84)/H-5 (*δ*_H_ 3.96)/H-6 (*δ*_H_ 1.87)/H-7 (*δ*_H_ 3.39)/H_2_-8 (*δ*_H_ 1.72, 1.40) (Figure 2).”

**FIGURE 2:**
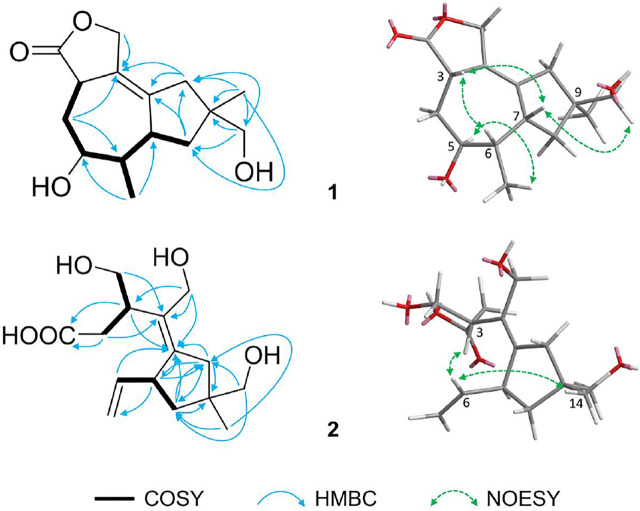
Key ^1^H-^1^H COSY (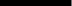), HMBC (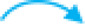) and NOESY (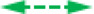) correlations of **1** and **2**.

On page 4, “Figure 1” is corrected to “Figure 2” as follows: “In addition, the relative configuration of **1** was determined by the NOESY correlations of H-3 (*δ*_H_ 3.62)/H-6 (*δ*_H_ 1.87)/H-7 (*δ*_H_ 3.39), and Me-13 (*δ*_H_ 0.90)/H-5 (*δ*_H_ 3.96)/Me-14 (*δ*_H_ 1.11) (Figure 2).”

“Figure 2” is corrected to “Figure 3” as follows: “Combined with the single-crystal X-ray diffraction analysis (Figure 3).”

**FIGURE 3:**
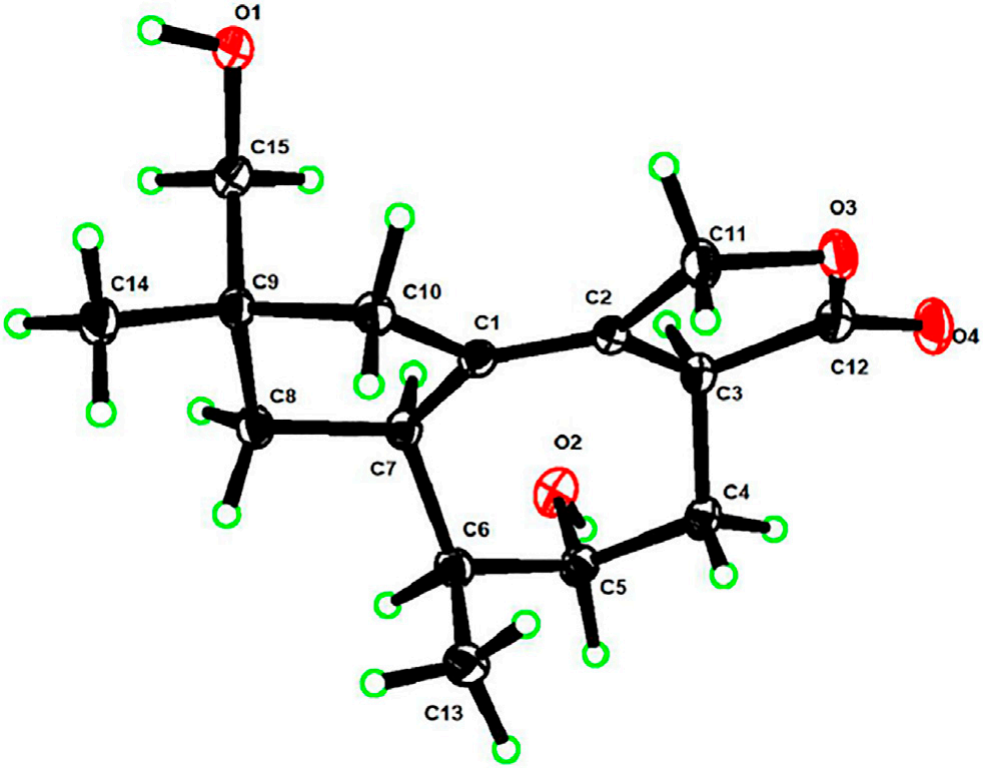
Perspective ORTEP drawing for **1**.

In the second paragraph, “Figure 3” is corrected to “Figure 1” as follows: “the planar structure of **2** was determined as shown in Figure 1.”

“Figure 1” is corrected to “Figure 2” as follows: “In addition, the relative configuration of **2** was confirmed by the NOESY correlations of H-6 (*δ*_H_ 5.81)/H-3 (*δ*_H_ 3.31)/Me-14 (*δ*_H_ 1.07) (Figure 2).”

The authors apologize for these errors and state that this does not change the scientific conclusions of the article in any way. The original article has been updated.

