# Supplementary material for: New Sesquiterpenoids From Plant-Associated Irpex lacteus
Source: Front Chem. 2022 May 17;10:905108. doi: 10.3389/fchem.2022.905108 (PMC9152251; doi:10.3389/fchem.2022.905108)
Supplement: Supplementary file 1 [file DataSheet1.docx]

**New Sesquiterpenoids from Plant-Associated *Irpex lacteus***

Huai-Zhi Luo^1,2^, Huan Jiang^2^, Xi-Shan Huang^3^, Ai-Qun Jia^1^*

^1^ *School of Pharmaceutical Sciences, Key Laboratory of Tropical Biological Resources of Ministry of Education, One Health Institute, Hainan University, Haikou 570228, China;*

^2^ *School of Environmental and Biological Engineering, Nanjing University of Science and Technology, Nanjing 210094, China;*

^3^ *State Key Laboratory for Chemistry and Molecular Engineering of Medicinal Resources, Collaborative Innovation Center for Guangxi Ethnic Medicine, School of Chemistry and Pharmaceutical Science, Guangxi Normal University, Guilin 541004, China.*

**^*^ Correspondence**

Corresponding Author:

Ai-Qun Jia. E-mail: ajia@hainanu.edu.cn. Tel: +86 898 66254967. Fax: +86 898 66254967. ORCID: 0000-0002-8089-6200.

**Contents**

[Figureure. S1.](#_Toc13288) ^[1](#_Toc13288)^[H NMR (MeOD, 400 MHz) of](#_Toc13288) **[1](#_Toc13288)** [1](#_Toc13288)

[Figureure. S2.](#_Toc31167) ^[13](#_Toc31167)^[C NMR (MeOD, 100 MHz) of](#_Toc31167) **[1](#_Toc31167)** [1](#_Toc31167)

[Figureure. S3. DEPT 135 and 90 spectra of](#_Toc12706) **[1](#_Toc12706)** [2](#_Toc12706)

[Figureure. S4. HSQC of](#_Toc22256) **[1](#_Toc22256)** [2](#_Toc22256)

[Figureure. S5.](#_Toc1170) ^[1](#_Toc1170)^[H-](#_Toc1170)^[1](#_Toc1170)^[H COSY of](#_Toc1170) **[1](#_Toc1170)** [3](#_Toc1170)

[Figure. S6. HMBC of](#_Toc21941) **[1](#_Toc21941)** [3](#_Toc21941)

[Figure. S7. NOESY of](#_Toc31777) **[1](#_Toc31777)** [4](#_Toc31777)

[Figure. S8. HRESIMS of](#_Toc914) **[1](#_Toc914)** [4](#_Toc914)

[Figure. S9. UV spectra of](#_Toc25750) **[1](#_Toc25750)** [5](#_Toc25750)

[Figure. S10. IR spectrum of](#_Toc25908) **[1](#_Toc25908)** [5](#_Toc25908)

[Figure. S11.](#_Toc6982) ^[1](#_Toc6982)^[H NMR (MeOD, 400 MHz) of](#_Toc6982) **[2](#_Toc6982)** [6](#_Toc6982)

[Figure. S12.](#_Toc10364) ^[13](#_Toc10364)^[C NMR (MeOD, 100 MHz) of](#_Toc10364) **[2](#_Toc10364)** [6](#_Toc10364)

[Figure. S13. DEPT 135 and 90 spectra of](#_Toc11526) **[2](#_Toc11526)** [7](#_Toc11526)

[Figure. S14. HSQC of](#_Toc11157) **[2](#_Toc11157)** [7](#_Toc11157)

[Figure. S15.](#_Toc6487) ^[1](#_Toc6487)^[H-](#_Toc6487)^[1](#_Toc6487)^[H COSY of](#_Toc6487) **[2](#_Toc6487)** [8](#_Toc6487)

[Figure. S16. HMBC of](#_Toc10737) **[2](#_Toc10737)** [8](#_Toc10737)

[Figure. S17. NOESY of](#_Toc26611) **[2](#_Toc26611)** [9](#_Toc26611)

[Figure. S18. HRESIMS of](#_Toc9778) **[2](#_Toc9778)** [9](#_Toc9778)

[Figure. S19. CD and UV spectra of](#_Toc27539) **[2](#_Toc27539)** [11](#_Toc27539)

[Figure. S20. IR spectrum of](#_Toc8258) **[2](#_Toc8258)** [11](#_Toc8258)

[Table S1.](#_Toc9266) ^[1](#_Toc9266)^[H (400 MHz) and](#_Toc9266) ^[13](#_Toc9266)^[C (100 MHz) NMR spectroscopic data of](#_Toc9266) **[1](#_Toc9266)** [and](#_Toc9266) **[2](#_Toc9266)** [(in CD3OD). 11](#_Toc9266)

[Figure. S21. B3LYP/6-31G(d) optimized low-energy conformers of](#_Toc24862) **[2](#_Toc24862)** [13](#_Toc24862)

[Table S2. The major conformers of](#_Toc1046) **[2](#_Toc1046)** [identified by conformational searches in MMFF94 force field using the micromodel 13](#_Toc1046)

[Table S3. Cartesian coordinates for the low-energy optimized conformers of](#_Toc12844) **[2](#_Toc12844)** [at B3LYP/6-31+g (d, p) level of theory in CH](#_Toc12844)_[3](#_Toc12844)_[OH 13](#_Toc12844)


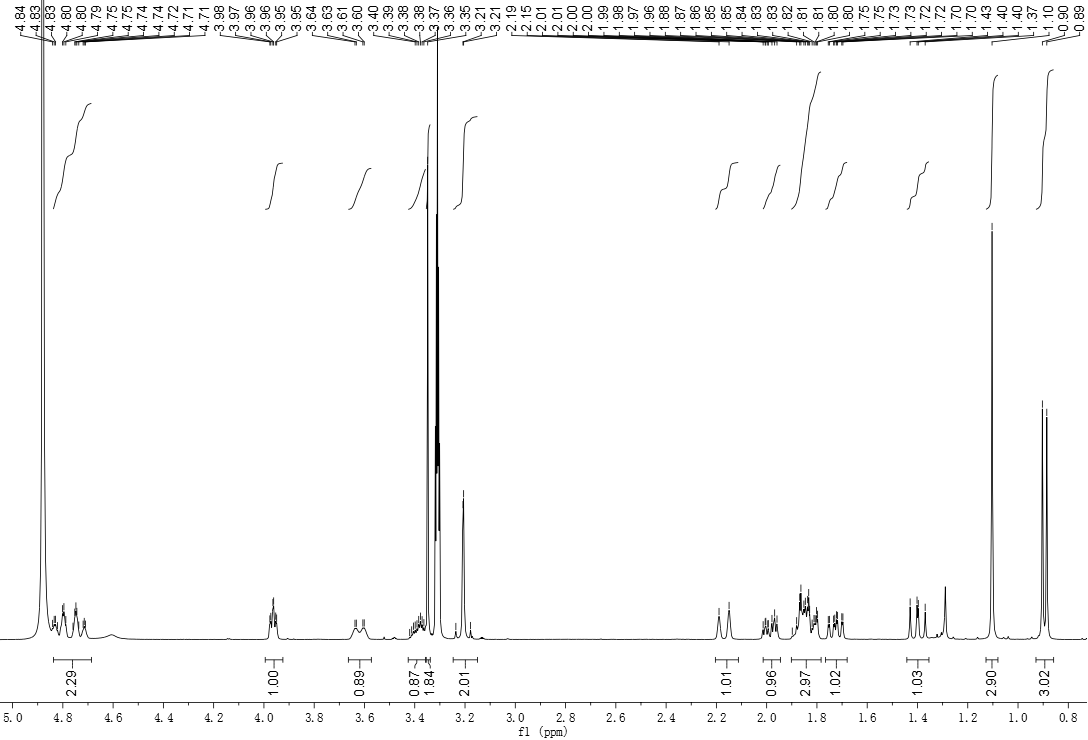


# Figure. S1. ^1^H NMR (MeOD, 400 MHz) of **1**


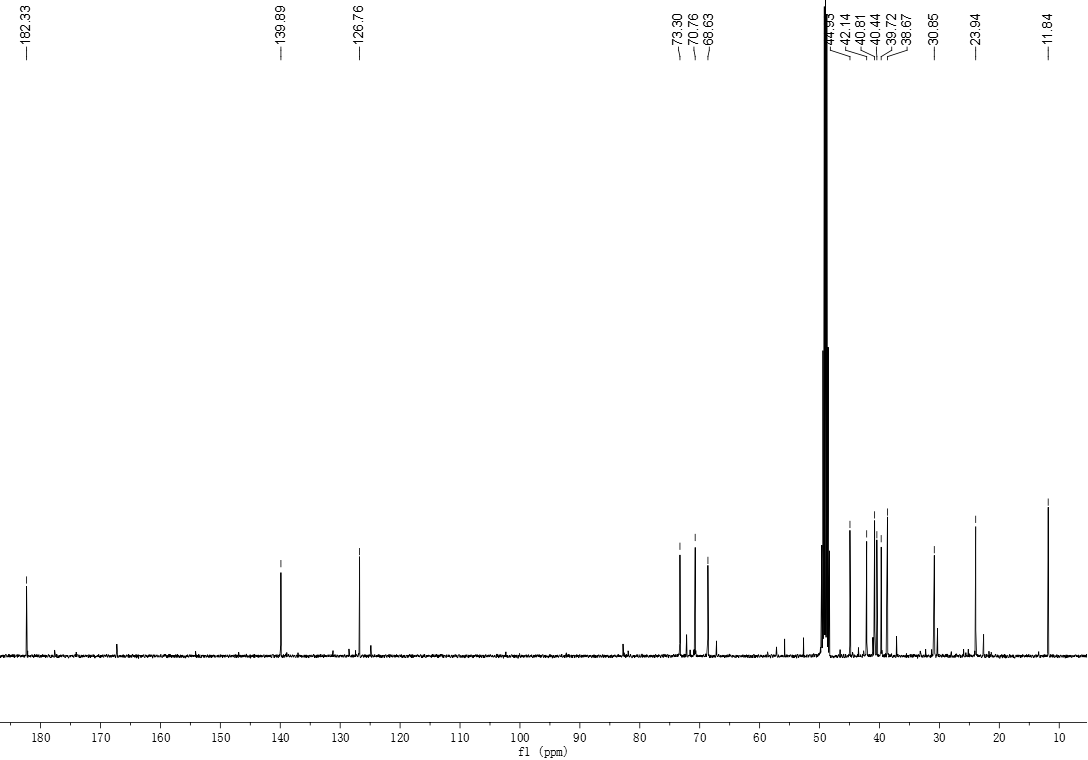


# Figure. S2. ^13^C NMR (MeOD, 100 MHz) of **1**


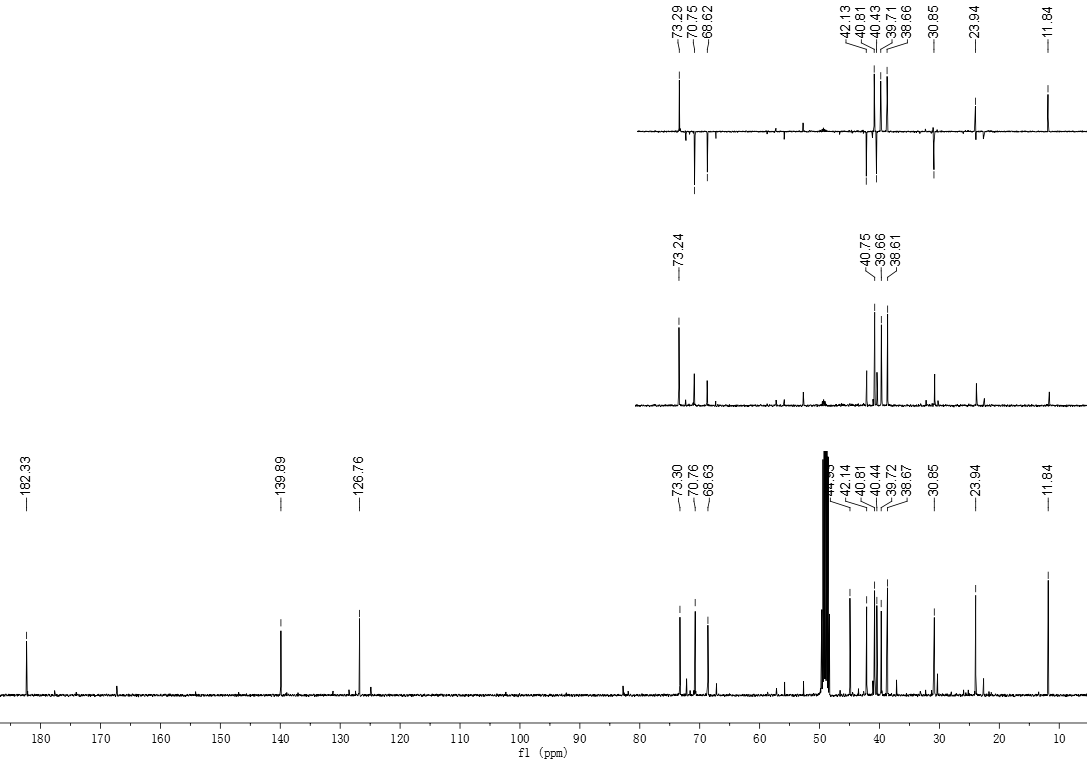


# Figure. S3. DEPT 135 and 90 spectra of **1**


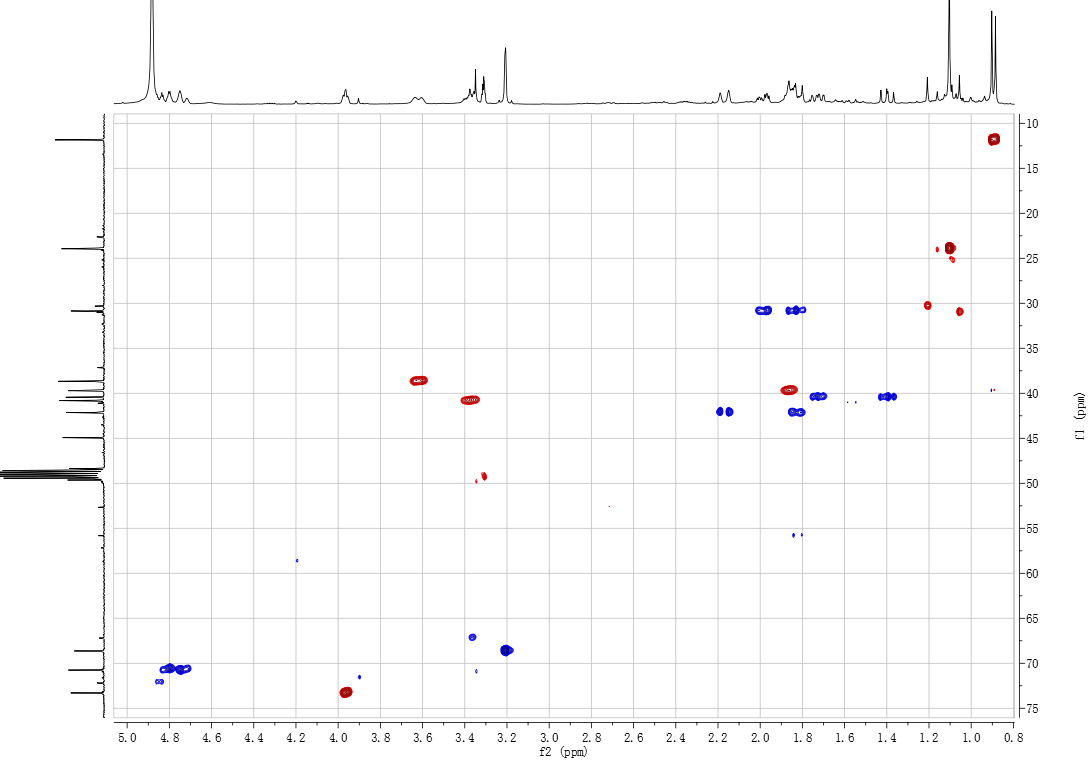


# Figure. S4. HSQC of **1**


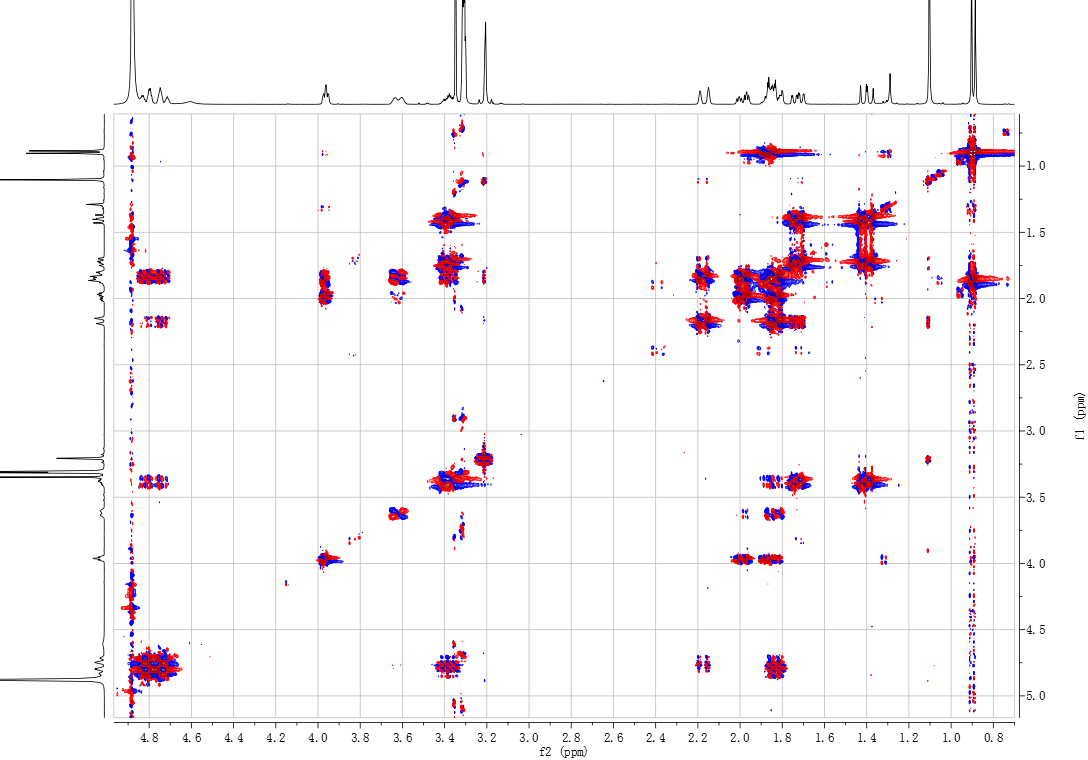


# Figure. S5. ^1^H-^1^H COSY of **1**


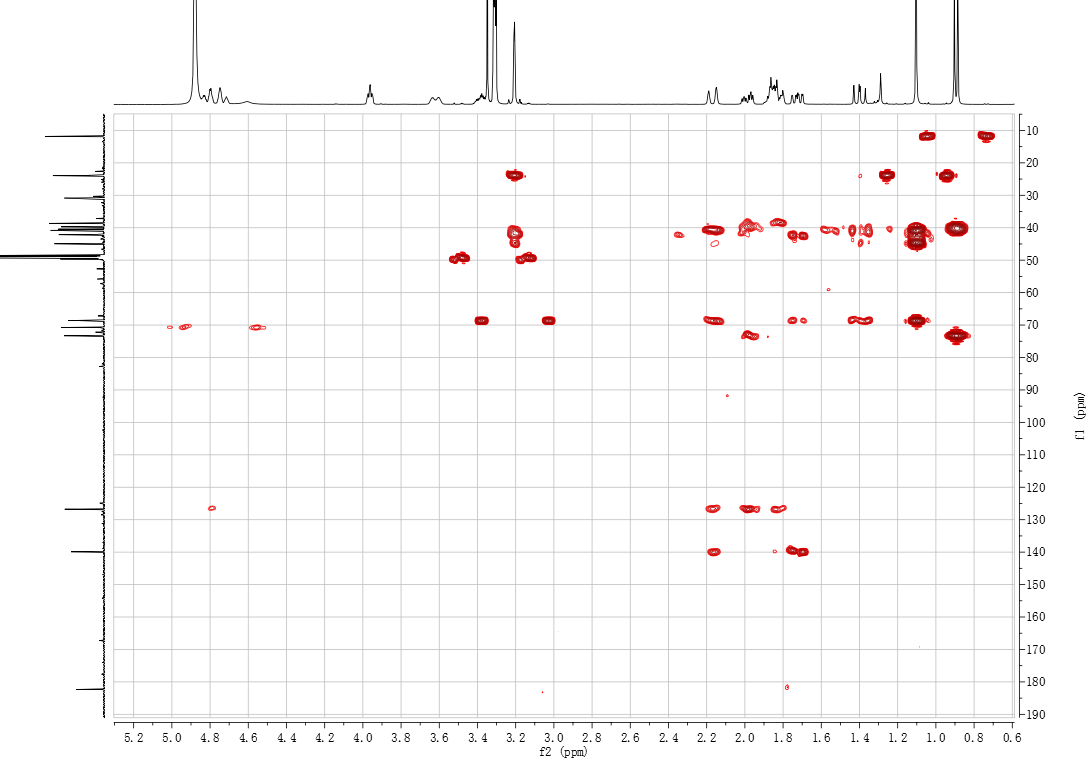


# Figure. S6. HMBC of **1**


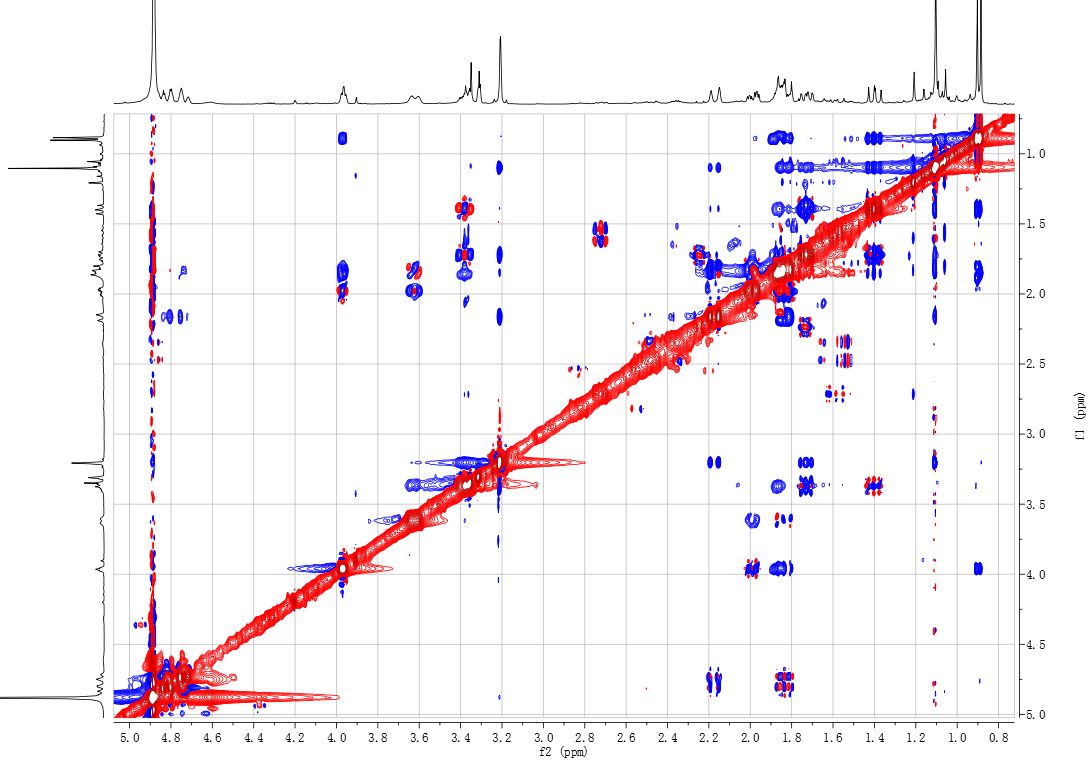


# Figure. S7. NOESY of **1**


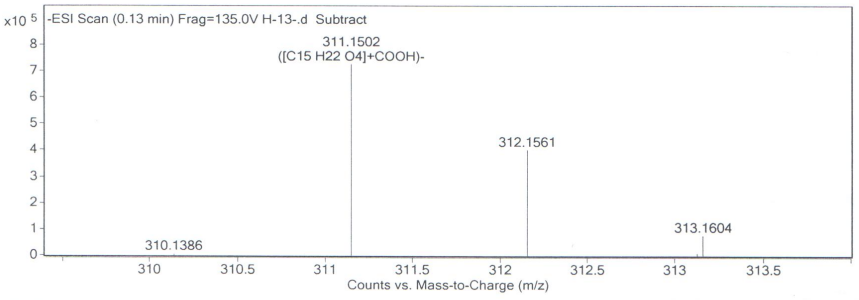

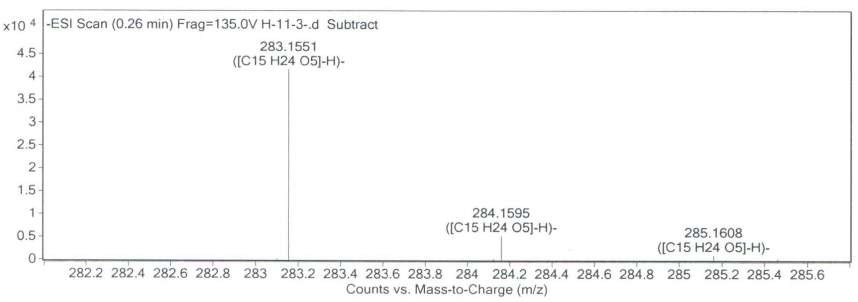


# Figure. S8. HRESIMS of **1**


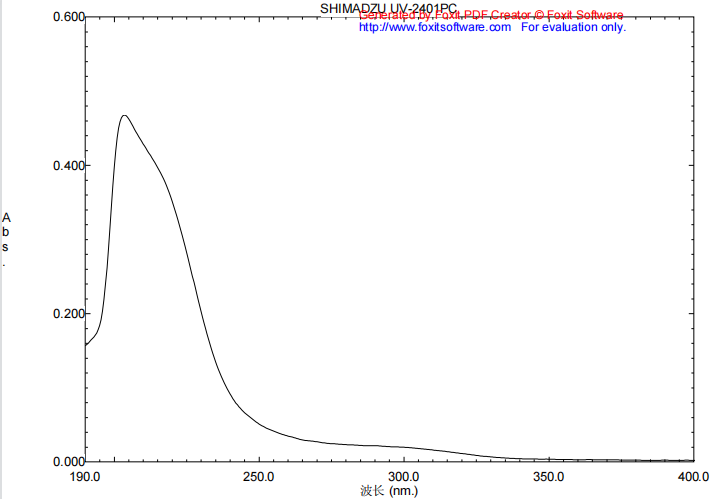


# Figure. S9. UV spectra of **1**


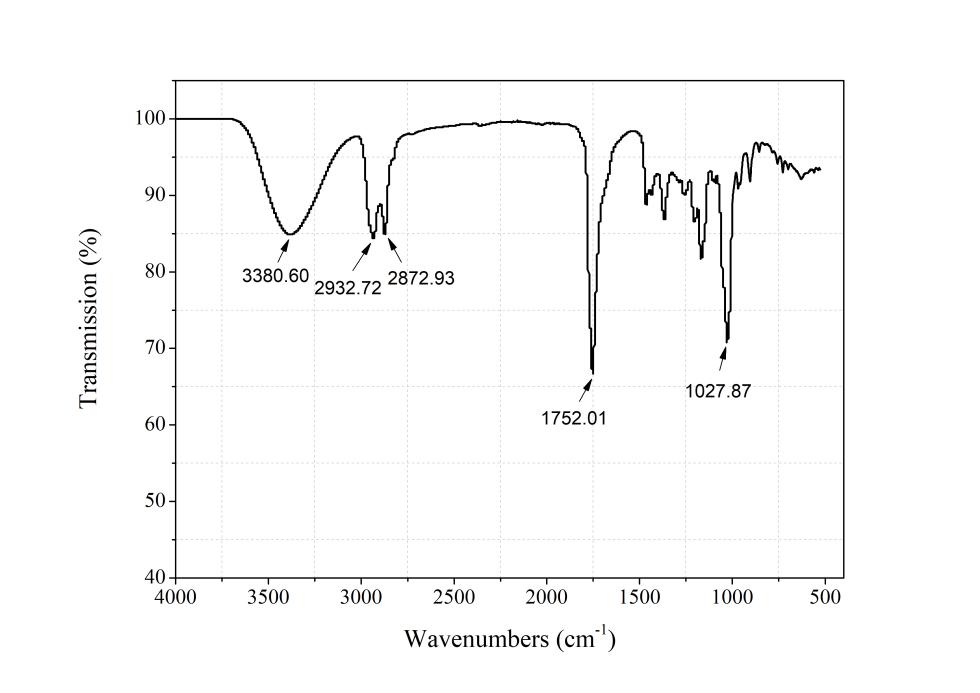


# Figure. S10. IR spectrum of **1**


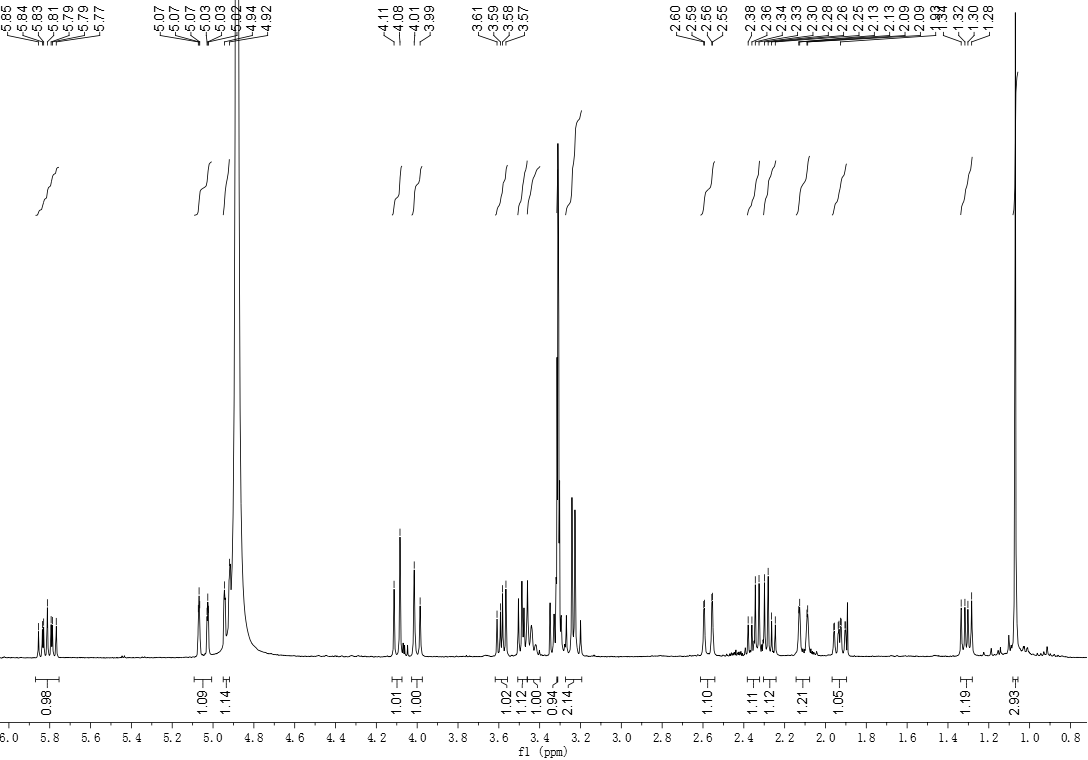


# Figure. S11. ^1^H NMR (MeOD, 400 MHz) of **2**


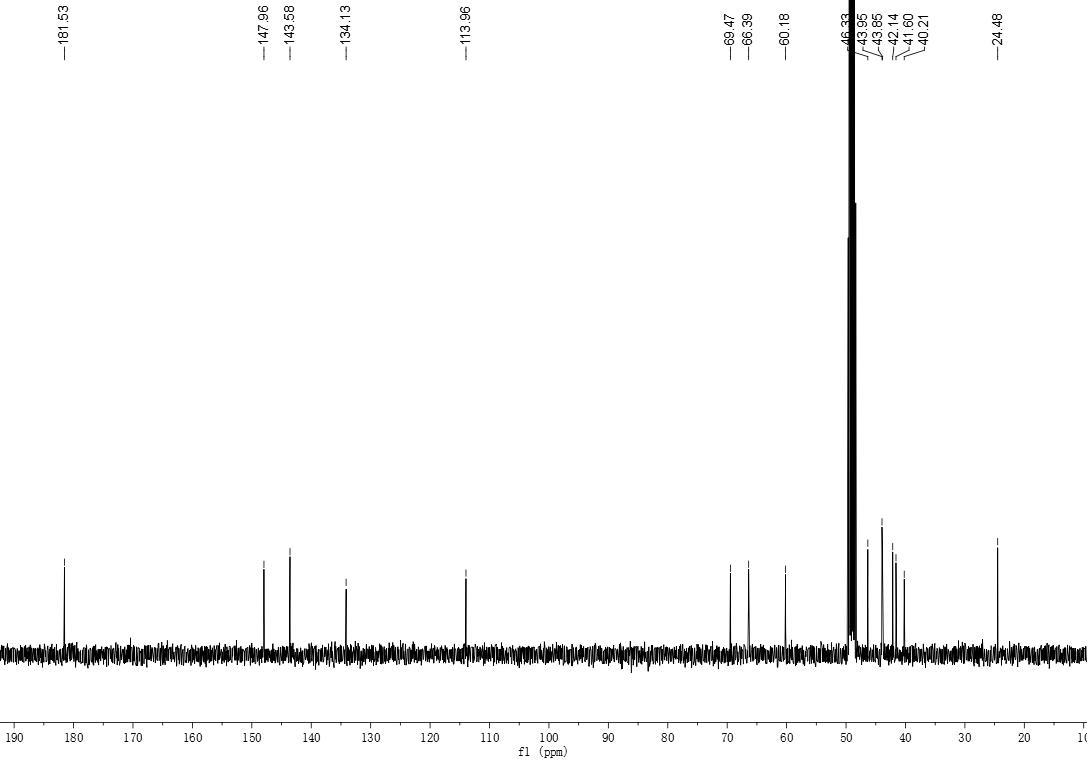


# Figure. S12. ^13^C NMR (MeOD, 100 MHz) of **2**


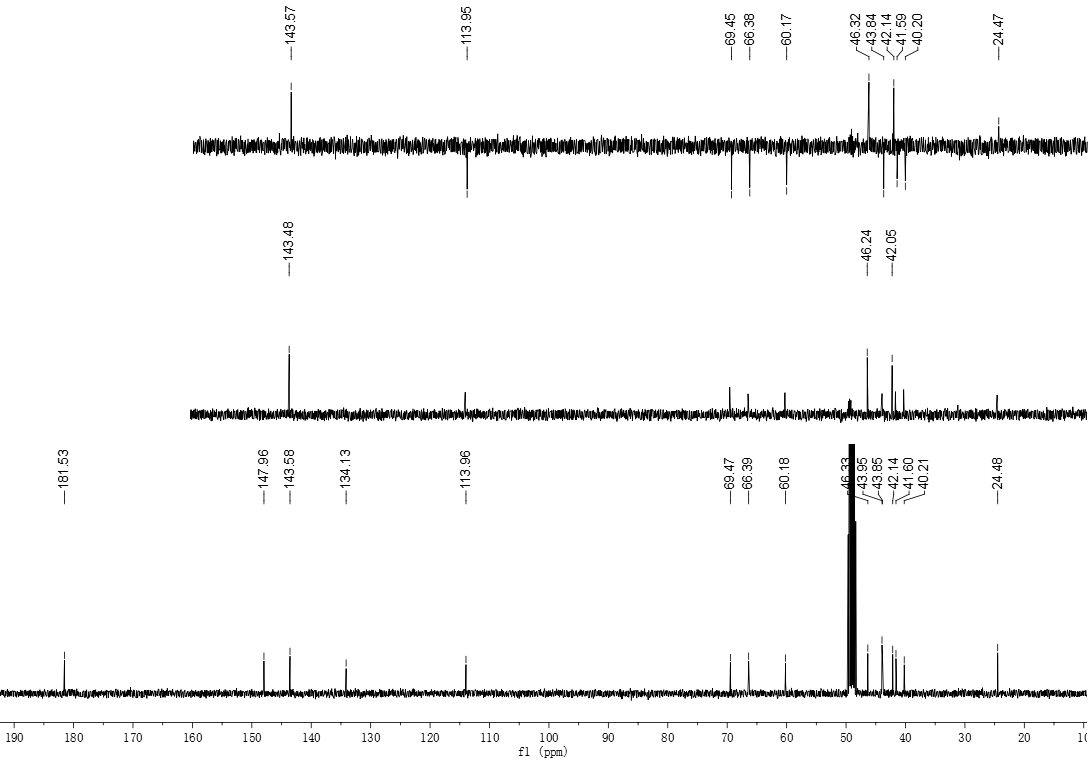


# Figure. S13. DEPT 135 and 90 spectra of **2**


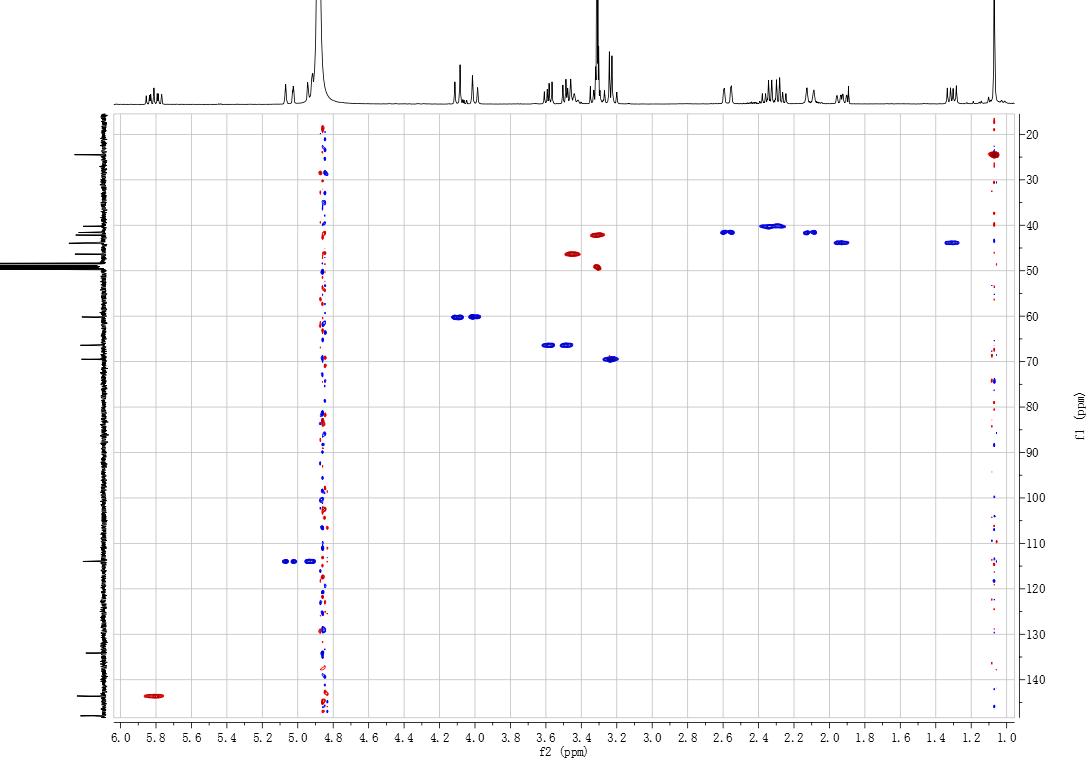


# Figure. S14. HSQC of **2**


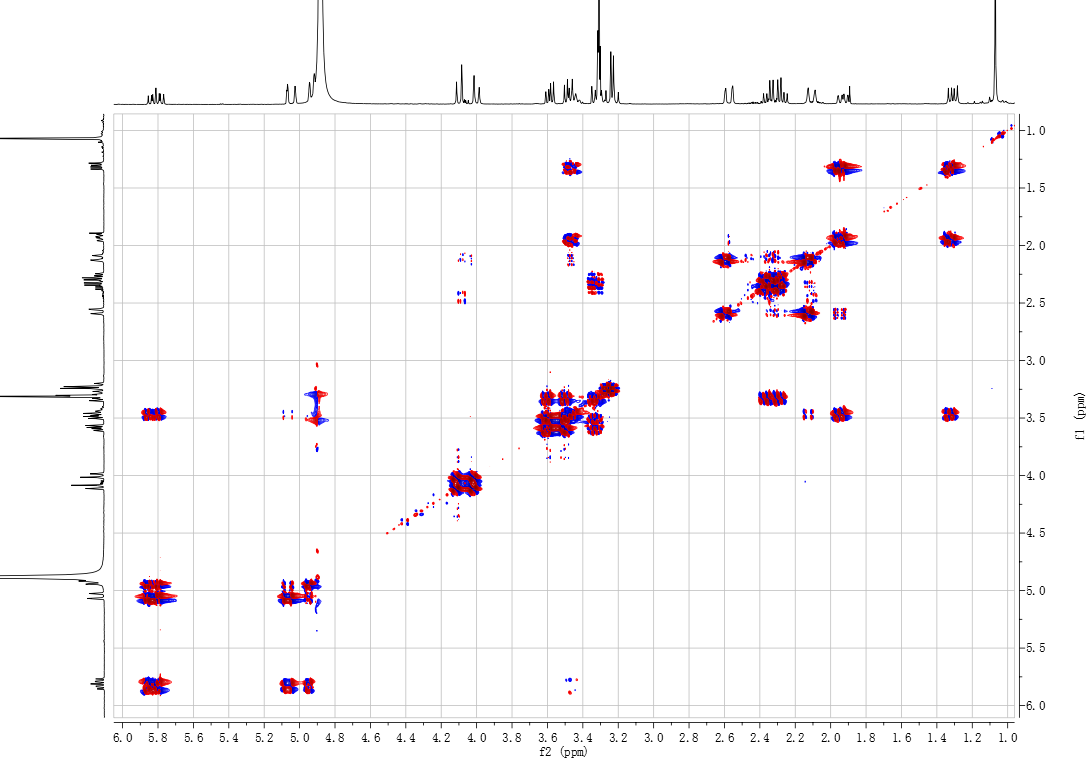


# Figure. S15. ^1^H-^1^H COSY of **2**


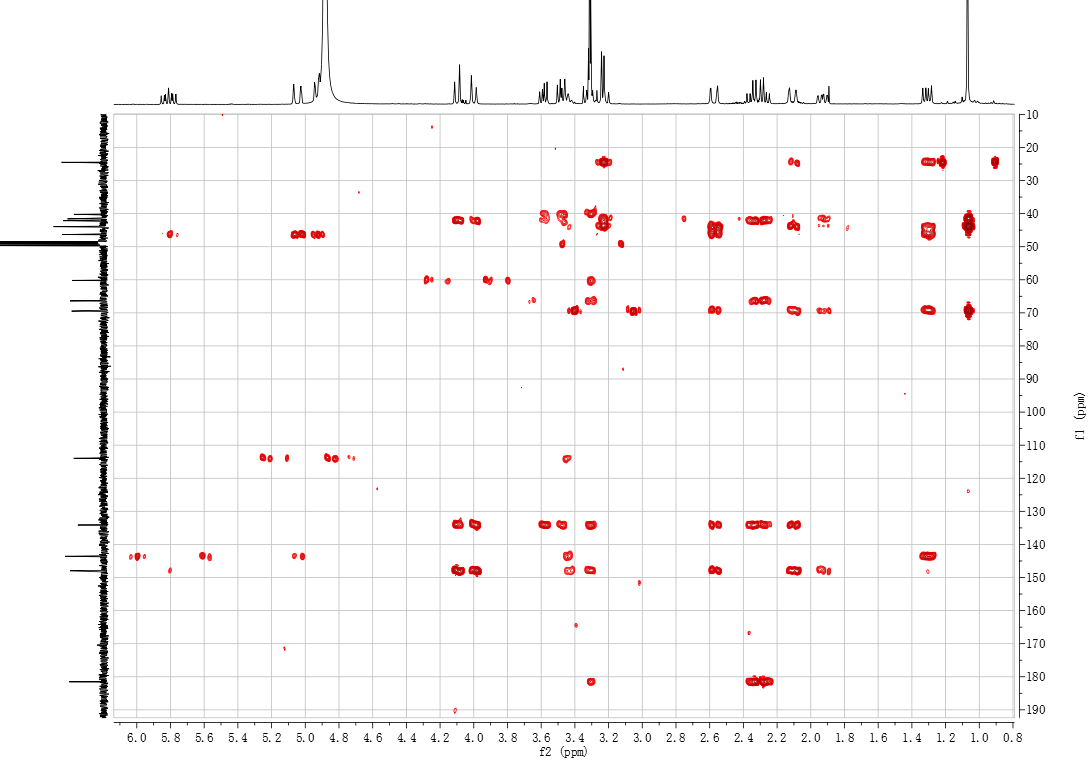


# Figure. S16. HMBC of **2**


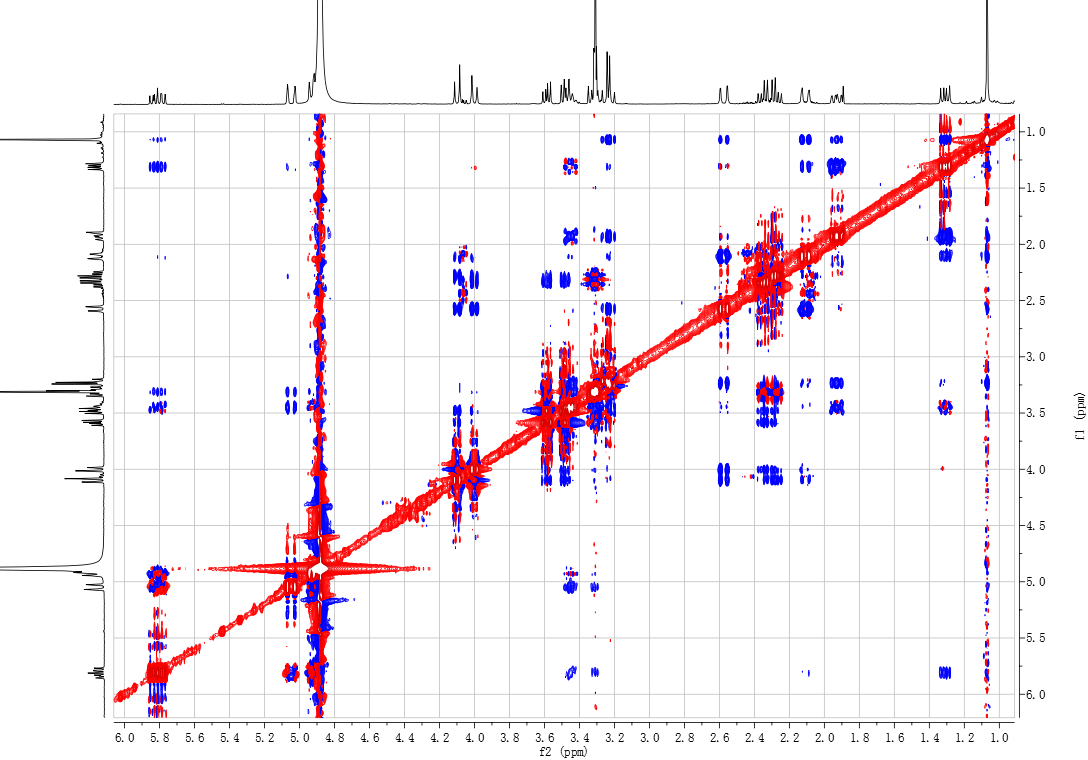


# Figure. S17. NOESY of **2**


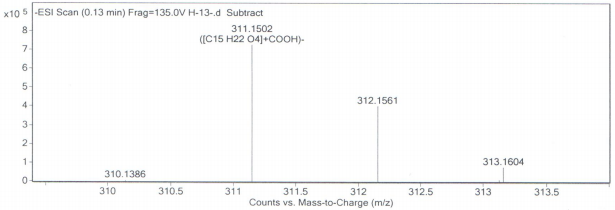


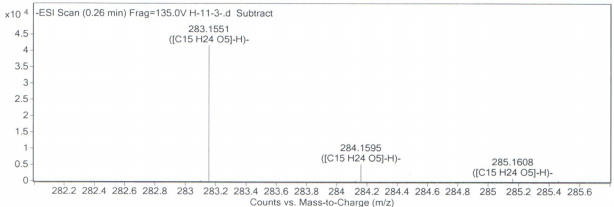


# Figure. S18. HRESIMS of **2**


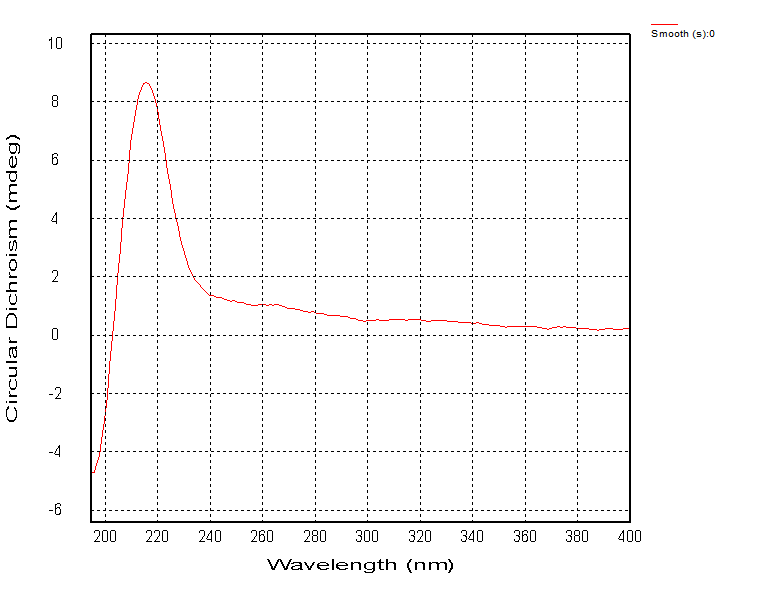

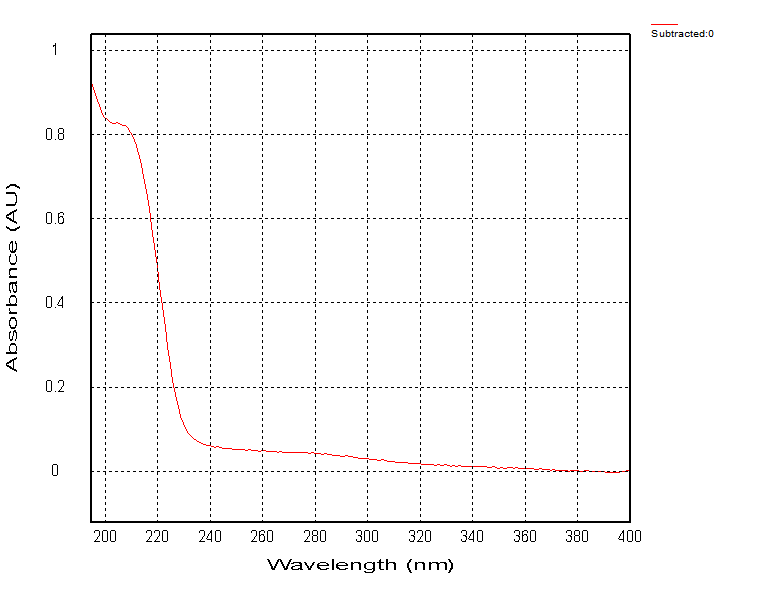


File: 1mm(195-400)21120313.dsx

ProBinaryX

Attributes :

- Time Stamp :Fri Dec 03 14:00:50 2021

- File ID : {6A34C66F-2222-4e32-BA93-36501A72DD8B}

- Is CFR Compliant : false

- Original data has not been modified.

Remarks:

- User: CD

- HV (CDDC channel): 0 v

- Time per point: 1 s

- Description: Sample 1

- Concentration: 0.6314mg/mL MeOH

- Pathlength: 1 mm

- Temperature: 20℃

Settings:

- Time-per-point: 1s (25us x 40000)

- SE

- Wavelength: 195nm - 400nm

- Step Size: 1nm

- Bandwidth: 1nm

# Figure. S19. CD and UV spectra of **2**


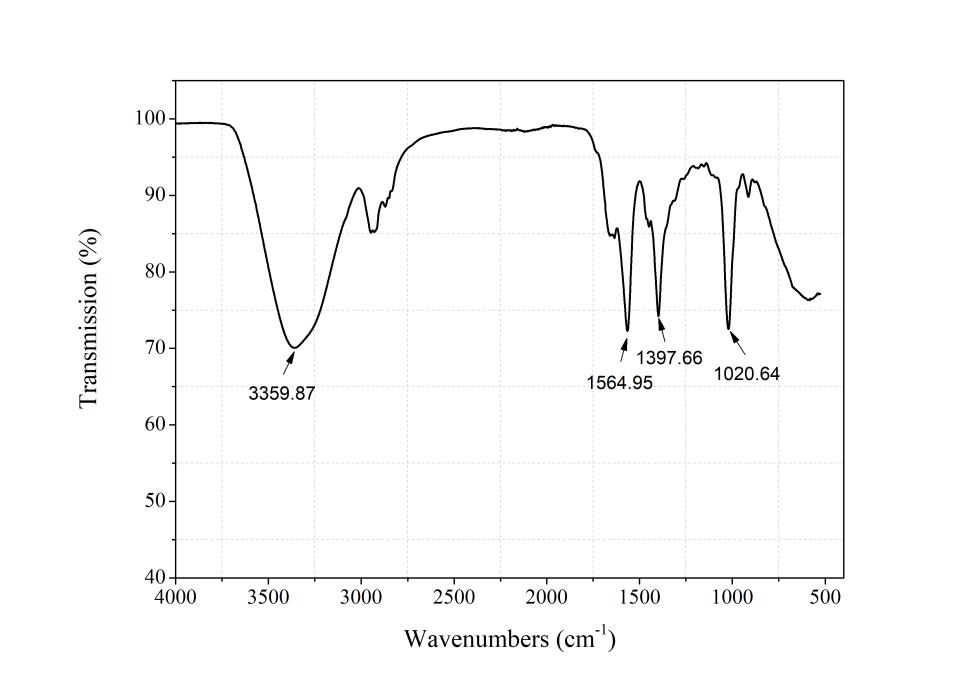


# Figure. S20. IR spectrum of **2**


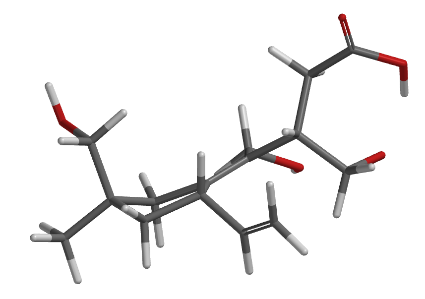

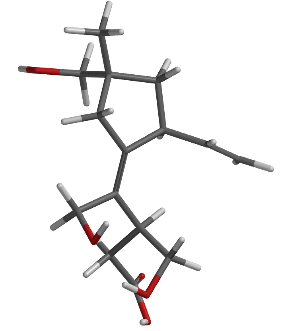


Conformer **2-1** 0 kJ/mol, 25.8% Conformer **2-2** 1.1 kJ/mol, 16.2%
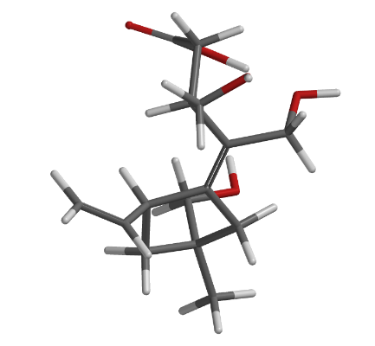

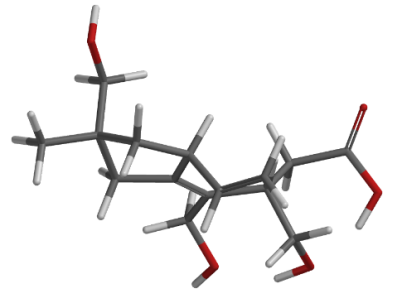


Conformer **2-3** 2.35 kJ/mol, 6.2% Conformer **2-4** 2.37 kJ/mol, 6.2%


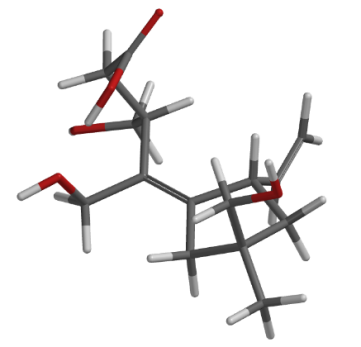

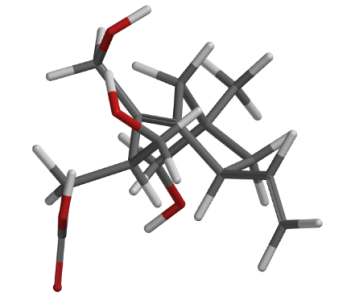


Conformer **2-5** 2.86 kJ/mol, 5.1% Conformer **2-6** 3.06 kJ/mol, 4.8%


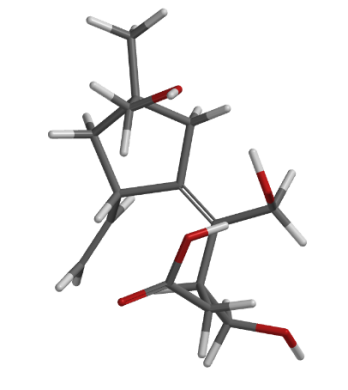

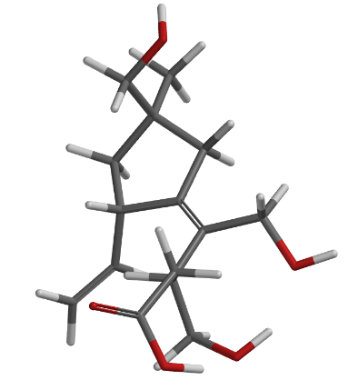


Conformer **2-7** 4.24 kJ/mol, 2.9% Conformer **2-8** 4.79kJ/mol, 2.3%


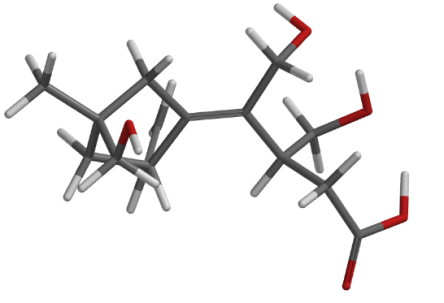

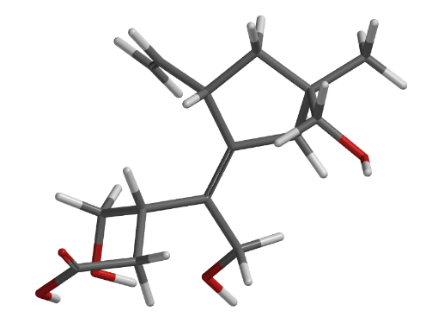


Conformer **2-9** 5.0 kJ/mol, 2.1% Conformer **2-10** 5.21 kJ/mol, 2.0%

# Figure. S21. B3LYP/6-31G(d) optimized low-energy conformers of **2**

# Table S1. The major conformers of **2** identified by conformational searches in MMFF94 force field using the micromodel

| Conformers | Δ*G^a^* | P (%)^b^ |
| --- | --- | --- |
| **2-1** | 0 | 25.8% |
| **2-2** | 1.10 | 16.2% |
| **2-3** | 2.35 | 6.2% |
| **2-4** | 2.37 | 6.2% |
| **2-5** | 2.86 | 5.1% |
| **2-6** | 3.06 | 4.8% |
| **2-7** | 4.24 | 2.9% |
| **2-8** | 4.79 | 2.3% |
| **2-9** | 5.00 | 2.1% |
| **2-10** | 5.21 | 2.0% |

*^a^ΔG*, B3LYP/6-31+g(d, p), in kj/mol. *^b^*Boltzmann-population

# Table S2. Cartesian coordinates for the low-energy optimized conformers of **2** at B3LYP/6-31+g (d, p) level of theory in CH_3_OH

Conformer **2-1**

| Atom | X | Y | Z |
| --- | --- | --- | --- |
| \| C \| \| --- \| \| C \| \| C \| \| C \| \| C \| \| C \| \| C \| \| O \| \| C \| \| C \| \| C \| \| C \| \| O \| \| C \| \| C \| \| C \| \| O \| \| C \| \| O \| \| O \| \| H \| \| H \| \| H \| \| H \| \| H \| \| H \| \| H \| \| H \| \| H \| \| H \| \| H \| \| H \| \| H \| \| H \| \| H \| \| H \| \| H \| \| H \| \| H \| \| H \| \| H \| \| H \| \| H \| \| H \| | \| 0 \| \| --- \| \| 0 \| \| 1.50063 \| \| 2.135365 \| \| 1.169811 \| \| -0.67881 \| \| -0.70054 \| \| -0.60576 \| \| 3.522954 \| \| 4.646984 \| \| 1.252131 \| \| 0.190215 \| \| 0.795628 \| \| 2.380279 \| \| 1.899268 \| \| 3.550874 \| \| 3.256954 \| \| 1.739619 \| \| 0.840461 \| \| 2.719329 \| \| 2.828753 \| \| -0.96181 \| \| 0.181548 \| \| 1.713392 \| \| 1.904104 \| \| 2.154321 \| \| -0.24903 \| \| -1.74731 \| \| -0.58308 \| \| -0.24313 \| \| -1.76027 \| \| -1.05476 \| \| 3.603012 \| \| 4.625633 \| \| 5.61923 \| \| -0.43545 \| \| -0.47797 \| \| \| 0.561711 \| \| --- \| \| 0.952372 \| \| 2.621652 \| \| 3.871458 \| \| 4.411864 \| \| 2.487452 \| \| 2.572569 \| \| | \| 0 \| \| --- \| \| 0 \| \| 0 \| \| 0.931218 \| \| 0.890982 \| \| 1.266867 \| \| -1.25239 \| \| -1.29479 \| \| 0.447836 \| \| 1.098616 \| \| 1.592004 \| \| 1.442606 \| \| 1.202084 \| \| 2.616752 \| \| 4.019681 \| \| 2.063737 \| \| 1.935544 \| \| 4.246747 \| \| 3.81421 \| \| 5.013161 \| \| 2.813349 \| \| 0.360114 \| \| -1.00741 \| \| 0.346296 \| \| -1.01903 \| \| 1.950969 \| \| 2.187258 \| \| 1.271935 \| \| 1.318999 \| \| -2.16626 \| \| -1.25593 \| \| -2.10663 \| \| -0.49951 \| \| 2.040431 \| \| 0.692956 \| \| 2.340967 \| \| 0.597132 \| \| 1.984552 \| \| 4.28114 \| \| 4.761618 \| \| 1.080969 \| \| 2.73593 \| \| 1.325736  4.980834 \| | \| 0 \| \| --- \| \| 1.535846 \| \| 1.851749 \| \| 0.803563 \| \| -0.38374 \| \| 2.097398 \| \| 2.082989 \| \| 3.500629 \| \| 0.472499 \| \| 0.801357 \| \| -1.54871 \| \| -2.619 \| \| -3.88038 \| \| -1.80679 \| \| -2.232 \| \| -2.65033 \| \| -4.03226 \| \| -3.71013 \| \| -4.41553 \| \| -4.22551 \| \| -0.82493 \| \| -0.37746 \| \| -0.39579 \| \| 2.870765 \| \| 1.771345 \| \| 1.20919 \| \| 1.689067 \| \| 1.854972 \| \| 3.187465 \| \| 1.686369 \| \| 1.806257 \| \| 3.794016 \| \| -0.05878 \| \| 1.339477 \| \| 0.538326 \| \| -2.6659 \| \| -2.44577 \| \| -4.4289 \| \| -1.74522 \| \| -1.86343 \| \| -2.28975 \| \| -2.5653 \| \| -4.08715 \| \| -5.19356 \| |

Conformer **2-2**

| Atom | X | Y | Z |
| --- | --- | --- | --- |
| \| C \| \| --- \| \| C \| \| C \| \| C \| \| C \| \| C \| \| C \| \| O \| \| C \| \| C \| \| C \| \| C \| \| O \| \| C \| \| C \| \| C \| \| O \| \| C \| \| O \| \| O \| \| H \| \| H \| \| H \| \| H \| \| H \| \| H \| \| H \| \| H \| \| H \| \| H \| \| H \| \| H \| \| H \| \| H \| \| H \| \| H \| \| H \| \| H \| \| H \| \| H \| \| H \| \| H \| \| H \| \| H \| | \| 0 \| \| --- \| \| 0 \| \| 1.49897 \| \| 2.140251 \| \| 1.171307 \| \| -0.6858 \| \| -0.68953 \| \| -2.04537 \| \| 3.519962 \| \| 4.653391 \| \| 1.262343 \| \| 0.228382 \| \| 0.875449 \| \| 2.394121 \| \| 1.856629 \| \| 3.489401 \| \| 3.231481 \| \| 2.942606 \| \| 3.127429 \| \| 3.696553 \| \| 2.880678 \| \| -0.95725 \| \| 0.182924 \| \| 1.70708 \| \| 1.90409 \| \| 2.175104 \| \| -0.21118 \| \| -1.73866 \| \| -0.64825 \| \| -0.66692 \| \| -0.1938 \| \| -2.44825 \| \| 3.585866 \| \| 4.646966 \| \| 5.619415 \| \| -0.39392 \| \| -0.4315 \| \| 0.172122 \| \| 1.347031 \| \| 1.106971 \| \| 3.586192 \| \| 4.462349 \| \| 2.429159 \| \| 3.503382 \| \|  \| | \| 0 \| \| --- \| \| 0 \| \| 0 \| \| 0.923493 \| \| 0.890527 \| \| 1.267587 \| \| -1.25035 \| \| -1.30225 \| \| 0.423983 \| \| 1.077539 \| \| 1.594552 \| \| 1.442112 \| \| 1.234475 \| \| 2.612878 \| \| 3.965194 \| \| 2.027188 \| \| 2.357788 \| \| 5.00637 \| \| 5.882768 \| \| 4.890452 \| \| 2.821144 \| \| 0.362325 \| \| -1.00768 \| \| 0.352727 \| \| -1.01918 \| \| 1.944003 \| \| 2.188178 \| \| 1.311169 \| \| 1.282058 \| \| -1.2453 \| \| -2.16541 \| \| -2.07801 \| \| -0.53671 \| \| 2.037402 \| \| 0.662799 \| \| 2.340324 \| \| 0.585079 \| \| 0.95238 \| \| 3.899336 \| \| 4.351976 \| \| 0.939757 \| \| 2.461232 \| \| 1.82517 \| \| 4.06609 \| | \| 0 \| \| --- \| \| 1.536602 \| \| 1.85682 \| \| 0.804833 \| \| -0.37734 \| \| 2.088973 \| \| 2.101042 \| \| 1.676943 \| \| 0.458418 \| \| 0.746479 \| \| -1.53531 \| \| -2.63368 \| \| -3.88614 \| \| -1.7803 \| \| -2.27862 \| \| -2.69418 \| \| -4.06382 \| \| -2.39193 \| \| -1.56081 \| \| -3.50704 \| \| -0.82054 \| \| -0.38676 \| \| -0.39358 \| \| 2.874511 \| \| 1.785545 \| \| 1.208547 \| \| 1.734504 \| \| 1.790126 \| \| 3.184064 \| \| 3.196628 \| \| 1.755611 \| \| 2.103841 \| \| -0.04992 \| \| 1.255559 \| \| 0.474271 \| \| -2.70513 \| \| -2.47639 \| \| -4.50034 \| \| -3.2475 \| \| -1.57545 \| \| -2.6029 \| \| -2.43933 \| \| -4.28359 \| \| -4.02048 \| |

Conformer **2-3**

| Atom | X | Y | Z |
| --- | --- | --- | --- |
| \| C \| \| --- \| \| C \| \| C \| \| C \| \| C \| \| C \| \| C \| \| O \| \| C \| \| C \| \| C \| \| C \| \| O \| \| C \| \| C \| \| C \| \| O \| \| C \| \| O \| \| O \| \| H \| \| H \| \| H \| \| H \| \| H \| \| H \| \| H \| \| H \| \| H \| \| H \| \| H \| \| H \| \| H \| \| H \| \| H \| \| H \| \| H \| \| H \| \| H \| \| H \| \| H \| \| H \| \| H \| \| H \| | \| 0 \| \| --- \| \| 0 \| \| 1.499423 \| \| 2.143563 \| \| 1.169743 \| \| -0.68404 \| \| -0.69048 \| \| -2.04442 \| \| 3.514581 \| \| 4.658339 \| \| 1.26435 \| \| 0.249841 \| \| 0.920261 \| \| 2.398589 \| \| 1.853711 \| \| 3.433998 \| \| 3.087043 \| \| 2.948776 \| \| 3.207528 \| \| 3.626917 \| \| 2.942875 \| \| -0.95944 \| \| 0.182807 \| \| 1.706544 \| \| 1.902366 \| \| 2.191899 \| \| -0.20727 \| \| -1.73637 \| \| -0.64716 \| \| -0.67411 \| \| -0.19371 \| \| -2.45391 \| \| 3.566221 \| \| 4.667156 \| \| 5.616746 \| \| -0.23046 \| \| -0.5532 \| \| 1.039781 \| \| 1.297411 \| \| 1.144767 \| \| 3.539783 \| \| 4.420165 \| \| 2.294367 \| \| 3.382466 \| | \| 0 \| \| --- \| \| 0 \| \| 0 \| \| 0.91745 \| \| 0.892074 \| \| 1.268466 \| \| -1.24941 \| \| -1.303 \| \| 0.398806 \| \| 1.041616 \| \| 1.601735 \| \| 1.452098 \| \| 0.986682 \| \| 2.620596 \| \| 4.008284 \| \| 2.090437 \| \| 2.49383 \| \| 5.040138 \| \| 5.841938 \| \| 4.998597 \| \| 2.76408 \| \| 0.360683 \| \| -1.00719 \| \| 0.355321 \| \| -1.02143 \| \| 1.936785 \| \| 2.188351 \| \| 1.314098 \| \| 1.282843 \| \| -1.24033 \| \| -2.16478 \| \| -2.07046 \| \| -0.56628 \| \| 2.004755 \| \| 0.613748 \| \| 2.407472 \| \| 0.743897 \| \| 0.028222 \| \| 4.010559 \| \| 4.357608 \| \| 1.000771 \| \| 2.515687 \| \| 1.950011 \| \| 4.221804 \| | \| 0 \| \| --- \| \| 1.537035 \| \| 1.857913 \| \| 0.802236 \| \| -0.37576 \| \| 2.08881 \| \| 2.101612 \| \| 1.670796 \| \| 0.445639 \| \| 0.718389 \| \| -1.53057 \| \| -2.64445 \| \| -3.81134 \| \| -1.77452 \| \| -2.15726 \| \| -2.78726 \| \| -4.11716 \| \| -2.26027 \| \| -1.37566 \| \| -3.42801 \| \| -0.83553 \| \| -0.38313 \| \| -0.39462 \| \| 2.873921 \| \| 1.791848 \| \| 1.205621 \| \| 1.734139 \| \| 1.789777 \| \| 3.183628 \| \| 3.196779 \| \| 1.7615 \| \| 2.107144 \| \| -0.05476 \| \| 1.22147 \| \| 0.442443 \| \| -2.87279 \| \| -2.4284 \| \| -3.67627 \| \| -3.10115 \| \| -1.39458 \| \| -2.75674 \| \| -2.57138 \| \| -4.34287 \| \| -3.99209 \| |

Conformer **2-4**

| Atom | X | Y | Z |
| --- | --- | --- | --- |
| \| C \| \| --- \| \| C \| \| C \| \| C \| \| C \| \| C \| \| C \| \| O \| \| C \| \| C \| \| C \| \| C \| \| O \| \| C \| \| C \| \| C \| \| O \| \| C \| \| O \| \| O \| \| H \| \| H \| \| H \| \| H \| \| H \| \| H \| \| H \| \| H \| \| H \| \| H \| \| H \| \| H \| \| H \| \| H \| \| H \| \| H \| \| H \| \| H \| \| H \| \| H \| \| H \| \| H \| \| H \| \| H \| | \| 0 \| \| --- \| \| 0 \| \| 1.504715 \| \| 2.134778 \| \| 1.154305 \| \| -0.67548 \| \| -0.71767 \| \| -0.16845 \| \| 3.510195 \| \| 4.646949 \| \| 1.237109 \| \| 0.227502 \| \| 0.906507 \| \| 2.357782 \| \| 1.792102 \| \| 3.400621 \| \| 3.048846 \| \| 2.870426 \| \| 3.111332 \| \| 3.553897 \| \| 2.898513 \| \| -0.96655 \| \| 0.197117 \| \| 1.718853 \| \| 1.918169 \| \| 2.174175 \| \| -0.21979 \| \| -1.73692 \| \| -0.6081 \| \| -1.78241 \| \| -0.62689 \| \| -0.62584 \| \| 3.568205 \| \| 4.648659 \| \| 5.608591 \| \| -0.26292 \| \| -0.56765 \| \| 1.077037 \| \| 1.237187 \| \| 1.07704 \| \| 3.522796 \| \| 4.380383 \| \| 2.268306 \| \| 3.323963 \| | \| 0 \| \| --- \| \| 0 \| \| 0 \| \| 0.934893 \| \| 0.911706 \| \| 1.268309 \| \| -1.22803 \| \| -2.4387 \| \| 0.437641 \| \| 1.098434 \| \| 1.625003 \| \| 1.459243 \| \| 0.992315 \| \| 2.659792 \| \| 4.039016 \| \| 2.143256 \| \| 2.542241 \| \| 5.087817 \| \| 5.898777 \| \| 5.053705 \| \| 2.811346 \| \| 0.348644 \| \| -0.99738 \| \| 0.346563 \| \| -1.01445 \| \| 1.95151 \| \| 2.189757 \| \| 1.294611 \| \| 1.293364 \| \| -1.21154 \| \| -1.24363 \| \| -3.1706 \| \| -0.53223 \| \| 2.063877 \| \| 0.67968 \| \| 2.406802 \| \| 0.743337 \| \| 0.045129 \| \| 4.032148 \| \| 4.377253 \| \| 1.055955 \| \| 2.584017 \| \| 1.981297 \| \| 4.271919 \| | \| 0 \| \| --- \| \| 1.538808 \| \| 1.844978 \| \| 0.796083 \| \| -0.3764 \| \| 2.100358 \| \| 2.120982 \| \| 1.617127 \| \| 0.428341 \| \| 0.684446 \| \| -1.52882 \| \| -2.64487 \| \| -3.80676 \| \| -1.7718 \| \| -2.15605 \| \| -2.78371 \| \| -4.11372 \| \| -2.2595 \| \| -1.37767 \| \| -3.42486 \| \| -0.83253 \| \| -0.37631 \| \| -0.40906 \| \| 2.862932 \| \| 1.764857 \| \| 1.209282 \| \| 1.722491 \| \| 1.830169 \| \| 3.193343 \| \| 1.863163 \| \| 3.213193 \| \| 2.068177 \| \| -0.06368 \| \| 1.18044 \| \| 0.401247 \| \| -2.88064 \| \| -2.42642 \| \| -3.64225 \| \| -3.10129 \| \| -1.39521 \| \| -2.75366 \| \| -2.56822 \| \| -4.34236 \| \| -3.98668 \| |

Conformer **2-5**

| Atom | X | Y | Z |
| --- | --- | --- | --- |
| \| C \| \| --- \| \| C \| \| C \| \| C \| \| C \| \| C \| \| C \| \| O \| \| C \| \| C \| \| C \| \| C \| \| O \| \| C \| \| C \| \| C \| \| O \| \| C \| \| O \| \| O \| \| H \| \| H \| \| H \| \| H \| \| H \| \| H \| \| H \| \| H \| \| H \| \| H \| \| H \| \| H \| \| H \| \| H \| \| H \| \| H \| \| H \| \| H \| \| H \| \| H \| \| H \| \| H \| \| H \| \| H \| | \| 0 \| \| --- \| \| 0 \| \| 1.504991 \| \| 2.132058 \| \| 1.152995 \| \| -0.6758 \| \| -0.71702 \| \| -0.1691 \| \| 3.512251 \| \| 4.643039 \| \| 1.2259 \| \| 0.192071 \| \| 0.837204 \| \| 2.341564 \| \| 1.779379 \| \| 3.441988 \| \| 3.174914 \| \| 2.845024 \| \| 3.012083 \| \| 3.601924 \| \| 2.828805 \| \| -0.96625 \| \| 0.200362 \| \| 1.720169 \| \| 1.920115 \| \| 2.162684 \| \| -0.2225 \| \| -1.73818 \| \| -0.60735 \| \| -1.7818 \| \| -0.62792 \| \| -0.62549 \| \| 3.579766 \| \| 4.634785 \| \| 5.609075 \| \| -0.45038 \| \| -0.44805 \| \| 0.14422 \| \| 1.26801 \| \| 1.025195 \| \| 3.552934 \| \| 4.409332 \| \| 2.380327 \| \| 3.424169 \| | \| 0 \| \| --- \| \| 0 \| \| 0 \| \| 0.940818 \| \| 0.914595 \| \| 1.268297 \| \| -1.22748 \| \| -2.4393 \| \| 0.457557 \| \| 1.123768 \| \| 1.635886 \| \| 1.478465 \| \| 1.303854 \| \| 2.673734 \| \| 4.024081 \| \| 2.116415 \| \| 2.465856 \| \| 5.08465 \| \| 5.957965 \| \| 4.996243 \| \| 2.877664 \| \| 0.347796 \| \| -0.99697 \| \| 0.342005 \| \| -1.0135 \| \| 1.955443 \| \| 2.190115 \| \| 1.294239 \| \| 1.294272 \| \| -1.2105 \| \| -1.2407 \| \| -3.16988 \| \| -0.50549 \| \| 2.084337 \| \| 0.715583 \| \| 2.362391 \| \| 0.60524 \| \| 0.989589 \| \| 3.96179 \| \| 4.386768 \| \| 1.029551 \| \| 2.56097 \| \| 1.923637 \| \| 4.17358 \| | \| 0 \| \| --- \| \| 1.539433 \| \| 1.843413 \| \| 0.797021 \| \| -0.37674 \| \| 2.100142 \| \| 2.121944 \| \| 1.622097 \| \| 0.432642 \| \| 0.702152 \| \| -1.52534 \| \| -2.62241 \| \| -3.88058 \| \| -1.76271 \| \| -2.24077 \| \| -2.68925 \| \| -4.05158 \| \| -2.34293 \| \| -1.50388 \| \| -3.45886 \| \| -0.80239 \| \| -0.38012 \| \| -0.40771 \| \| 2.862222 \| \| 1.756563 \| \| 1.213828 \| \| 1.721108 \| \| 1.83095 \| \| 3.193122 \| \| 1.863283 \| \| 3.214269 \| \| 2.073791 \| \| -0.07128 \| \| 1.208722 \| \| 0.41921 \| \| -2.67711 \| \| -2.47426 \| \| -4.49154 \| \| -3.20812 \| \| -1.53078 \| \| -2.6152 \| \| -2.42952 \| \| -4.27747 \| \| -3.98228 \| |

Conformer **2-6**

| Atom | X | Y | Z |
| --- | --- | --- | --- |
| \| C \| \| --- \| \| C \| \| C \| \| C \| \| C \| \| C \| \| C \| \| O \| \| C \| \| C \| \| C \| \| C \| \| O \| \| C \| \| C \| \| C \| \| O \| \| C \| \| O \| \| O \| \| H \| \| H \| \| H \| \| H \| \| H \| \| H \| \| H \| \| H \| \| H \| \| H \| \| H \| \| H \| \| H \| \| H \| \| H \| \| H \| \| H \| \| H \| \| H \| \| H \| \| H \| \| H \| \| H \| \| H \| | \| 0 \| \| --- \| \| 0 \| \| 1.501261 \| \| 2.140361 \| \| 1.168603 \| \| -0.67979 \| \| -0.68465 \| \| -2.04621 \| \| 3.518155 \| \| 4.653197 \| \| 1.257757 \| \| 0.22634 \| \| 0.877479 \| \| 2.38674 \| \| 1.844314 \| \| 3.484531 \| \| 3.227391 \| \| 2.924886 \| \| 3.102378 \| \| 3.683155 \| \| 2.872312 \| \| -0.95842 \| \| 0.183855 \| \| 1.709811 \| \| 1.905693 \| \| 2.176284 \| \| -0.18472 \| \| -1.72519 \| \| -0.66619 \| \| -0.64152 \| \| -0.19228 \| \| -2.55428 \| \| 3.582983 \| \| 4.64879 \| \| 5.618033 \| \| -0.39951 \| \| -0.42887 \| \| 0.181132 \| \| 1.337083 \| \| 1.090287 \| \| 3.582756 \| \| 4.455769 \| \| 2.42726 \| \| 3.495215 \| | \| 0 \| \| --- \| \| 0 \| \| 0 \| \| 0.924803 \| \| 0.89269 \| \| 1.269025 \| \| -1.25568 \| \| -1.32466 \| \| 0.425058 \| \| 1.07898 \| \| 1.597598 \| \| 1.438783 \| \| 1.232825 \| \| 2.618403 \| \| 3.969666 \| \| 2.038275 \| \| 2.372375 \| \| 5.015 \| \| 5.890767 \| \| 4.905976 \| \| 2.82705 \| \| 0.360308 \| \| -1.00712 \| \| 0.350568 \| \| -1.02002 \| \| 1.944267 \| \| 2.187274 \| \| 1.333922 \| \| 1.268069 \| \| -1.28064 \| \| -2.16347 \| \| -0.72557 \| \| -0.53656 \| \| 2.039264 \| \| 0.663467 \| \| 2.333519 \| \| 0.579243 \| \| 0.930375 \| \| 3.903157 \| \| 4.350208 \| \| 0.950549 \| \| 2.473492 \| \| 1.836888 \| \| 4.081899 \| | \| 0 \| \| --- \| \| 1.537184 \| \| 1.853851 \| \| 0.802138 \| \| -0.37788 \| \| 2.091435 \| \| 2.098035 \| \| 1.693599 \| \| 0.451913 \| \| 0.735798 \| \| -1.53607 \| \| -2.63689 \| \| -3.88734 \| \| -1.77985 \| \| -2.27753 \| \| -2.69572 \| \| -4.06439 \| \| -2.38524 \| \| -1.5505 \| \| -3.49798 \| \| -0.82062 \| \| -0.3865 \| \| -0.39492 \| \| 2.871793 \| \| 1.780038 \| \| 1.207162 \| \| 1.759665 \| \| 1.768998 \| \| 3.186818 \| \| 3.192435 \| \| 1.731887 \| \| 2.265913 \| \| -0.05546 \| \| 1.24438 \| \| 0.461681 \| \| -2.71008 \| \| -2.4797 \| \| -4.49947 \| \| -3.24742 \| \| -1.57618 \| \| -2.60884 \| \| -2.43797 \| \| -4.28738 \| \| -4.01461 \| |

Conformer **2-7**

| Atom | X | Y | Z |
| --- | --- | --- | --- |
| \| C \| \| --- \| \| C \| \| C \| \| C \| \| C \| \| C \| \| C \| \| O \| \| C \| \| C \| \| C \| \| C \| \| O \| \| C \| \| C \| \| C \| \| O \| \| C \| \| O \| \| O \| \| H \| \| H \| \| H \| \| H \| \| H \| \| H \| \| H \| \| H \| \| H \| \| H \| \| H \| \| H \| \| H \| \| H \| \| H \| \| H \| \| H \| \| H \| \| H \| \| H \| \| H \| \| H \| \| H \| \| H \| | \| 0 \| \| --- \| \| 0 \| \| 1.500363 \| \| 2.137915 \| \| 1.167979 \| \| -0.67974 \| \| -0.70146 \| \| -0.60625 \| \| 3.518987 \| \| 4.650326 \| \| 1.259012 \| \| 0.228315 \| \| 0.879443 \| \| 2.391872 \| \| 1.855802 \| \| 3.492055 \| \| 3.238881 \| \| 2.939899 \| \| 3.116181 \| \| 3.700076 \| \| 2.871516 \| \| -0.96008 \| \| 0.183467 \| \| 1.71229 \| \| 1.904389 \| \| 2.168078 \| \| -0.24676 \| \| -1.7474 \| \| -0.58668 \| \| -0.24553 \| \| -1.76249 \| \| -1.05735 \| \| 3.588387 \| \| 4.639088 \| \| 5.617572 \| \| -0.38922 \| \| -0.43707 \| \| 0.175747 \| \| 1.349989 \| \| 1.101834 \| \| 3.589872 \| \| 4.462675 \| \| 2.440124 \| \| 3.513395 \| | \| 0 \| \| --- \| \| 0 \| \| 0 \| \| 0.927516 \| \| 0.893451 \| \| 1.266971 \| \| -1.25174 \| \| -1.29481 \| \| 0.434041 \| \| 1.094038 \| \| 1.597765 \| \| 1.445366 \| \| 1.226253 \| \| 2.617615 \| \| 3.966825 \| \| 2.027907 \| \| 2.349524 \| \| 5.008958 \| \| 5.894855 \| \| 4.88643 \| \| 2.830866 \| \| 0.359706 \| \| -1.00598 \| \| 0.34937 \| \| -1.01902 \| \| 1.947588 \| \| 2.187072 \| \| 1.27392 \| \| 1.318046 \| \| -2.16554 \| \| -1.2547 \| \| -2.10454 \| \| -0.52873 \| \| 2.053254 \| \| 0.681619 \| \| 2.346 \| \| 0.594069 \| \| 0.966676 \| \| 3.894986 \| \| 4.356198 \| \| 0.941846 \| \| 2.466075 \| \| 1.811479 \| \| 4.056886 \| | \| 0 \| \| --- \| \| 1.53647 \| \| 1.855216 \| \| 0.804728 \| \| -0.37766 \| \| 2.097836 \| \| 2.084127 \| \| 3.500901 \| \| 0.456738 \| \| 0.740622 \| \| -1.53605 \| \| -2.6385 \| \| -3.88583 \| \| -1.77933 \| \| -2.28763 \| \| -2.6864 \| \| -4.05858 \| \| -2.40352 \| \| -1.57923 \| \| -3.5135 \| \| -0.81815 \| \| -0.38347 \| \| -0.39519 \| \| 2.872868 \| \| 1.778245 \| \| 1.210193 \| \| 1.691812 \| \| 1.852436 \| \| 3.18748 \| \| 1.686524 \| \| 1.808332 \| \| 3.797109 \| \| -0.0464 \| \| 1.248822 \| \| 0.47089 \| \| -2.71619 \| \| -2.47746 \| \| -4.50883 \| \| -3.2574 \| \| -1.59099 \| \| -2.58835 \| \| -2.42972 \| \| -4.27903 \| \| -4.02187 \| |

Conformer **2-8**

| Atom | X | Y | Z |
| --- | --- | --- | --- |
| \| C \| \| --- \| \| C \| \| C \| \| C \| \| C \| \| C \| \| C \| \| O \| \| C \| \| C \| \| C \| \| C \| \| O \| \| C \| \| C \| \| C \| \| O \| \| C \| \| O \| \| O \| \| H \| \| H \| \| H \| \| H \| \| H \| \| H \| \| H \| \| H \| \| H \| \| H \| \| H \| \| H \| \| H \| \| H \| \| H \| \| H \| \| H \| \| H \| \| H \| \| H \| \| H \| \| H \| \| H \| \| H \| | \| 0 \| \| --- \| \| 0 \| \| 1.499843 \| \| 2.142617 \| \| 1.16708 \| \| -0.68079 \| \| -0.68558 \| \| -2.04281 \| \| 3.513901 \| \| 4.656862 \| \| 1.260343 \| \| 0.249223 \| \| 0.923916 \| \| 2.392729 \| \| 1.842488 \| \| 3.431335 \| \| 3.087154 \| \| 2.933873 \| \| 3.184454 \| \| 3.618415 \| \| 2.93465 \| \| -0.96013 \| \| 0.186657 \| \| 1.70767 \| \| 1.903882 \| \| 2.191071 \| \| -0.18546 \| \| -1.72602 \| \| -0.66918 \| \| -0.65182 \| \| -0.18642 \| \| -2.55814 \| \| 3.564976 \| \| 4.66699 \| \| 5.615874 \| \| -0.23637 \| \| -0.55016 \| \| 1.043328 \| \| 1.291448 \| \| 1.129738 \| \| 3.539963 \| \| 4.416009 \| \| 2.298576 \| \| 3.379872 \| | \| 0 \| \| --- \| \| 0 \| \| 0 \| \| 0.919349 \| \| 0.896263 \| \| 1.268123 \| \| -1.2557 \| \| -1.33193 \| \| 0.403264 \| \| 1.047925 \| \| 1.605587 \| \| 1.451897 \| \| 0.991859 \| \| 2.628232 \| \| 4.012857 \| \| 2.100471 \| \| 2.504932 \| \| 5.049212 \| \| 5.852321 \| \| 5.011287 \| \| 2.772978 \| \| 0.358496 \| \| -1.00563 \| \| 0.355417 \| \| -1.01933 \| \| 1.938872 \| \| 2.186947 \| \| 1.334093 \| \| 1.266925 \| \| -1.27712 \| \| -2.16261 \| \| -0.72912 \| \| -0.56259 \| \| 2.010988 \| \| 0.620501 \| \| 2.404294 \| \| 0.739319 \| \| 0.032175 \| \| 4.012552 \| \| 4.358957 \| \| 1.011149 \| \| 2.527143 \| \| 1.956625 \| \| 4.233461 \| | \| 0 \| \| --- \| \| 1.537086 \| \| 1.856461 \| \| 0.802261 \| \| -0.37494 \| \| 2.092068 \| \| 2.096703 \| \| 1.680917 \| \| 0.442765 \| \| 0.713188 \| \| -1.52974 \| \| -2.64571 \| \| -3.81225 \| \| -1.77262 \| \| -2.15741 \| \| -2.78358 \| \| -4.11292 \| \| -2.25559 \| \| -1.36925 \| \| -3.41984 \| \| -0.83227 \| \| -0.38313 \| \| -0.39629 \| \| 2.873177 \| \| 1.788925 \| \| 1.205681 \| \| 1.761588 \| \| 1.768182 \| \| 3.187692 \| \| 3.192134 \| \| 1.739164 \| \| 2.243114 \| \| -0.05694 \| \| 1.214718 \| \| 0.435458 \| \| -2.87353 \| \| -2.43232 \| \| -3.68108 \| \| -3.10457 \| \| -1.39778 \| \| -2.75365 \| \| -2.56434 \| \| -4.34349 \| \| -3.98485 \| |

Conformer **2-9**

| Atom | X | Y | Z |
| --- | --- | --- | --- |
| \| C \| \| --- \| \| C \| \| C \| \| C \| \| C \| \| C \| \| C \| \| O \| \| C \| \| C \| \| C \| \| C \| \| O \| \| C \| \| C \| \| C \| \| O \| \| C \| \| O \| \| O \| \| H \| \| H \| \| H \| \| H \| \| H \| \| H \| \| H \| \| H \| \| H \| \| H \| \| H \| \| H \| \| H \| \| H \| \| H \| \| H \| \| H \| \| H \| \| H \| \| H \| \| H \| \| H \| \| H \| \| H \| | \| 0 \| \| --- \| \| 0 \| \| 1.498505 \| \| 2.152224 \| \| 1.153322 \| \| -0.68438 \| \| -0.69014 \| \| -2.04712 \| \| 3.53371 \| \| 3.899982 \| \| 1.206991 \| \| 0.141734 \| \| 0.744578 \| \| 2.334526 \| \| 1.764791 \| \| 3.241394 \| \| 2.738394 \| \| 2.850542 \| \| 3.229113 \| \| 3.373483 \| \| 2.995287 \| \| -0.96816 \| \| 0.202642 \| \| 1.704527 \| \| 1.896587 \| \| 2.239741 \| \| -0.20053 \| \| -1.73425 \| \| -0.65815 \| \| -0.66865 \| \| -0.19657 \| \| -2.45233 \| \| 4.302037 \| \| 3.191363 \| \| 4.931733 \| \| -0.33403 \| \| -0.66074 \| \| 0.895077 \| \| 1.094031 \| \| 1.16309 \| \| 3.355509 \| \| 4.246205 \| \| 1.964677 \| \| 3.051842 \| | \| 0 \| \| --- \| \| 0 \| \| 0 \| \| 0.894378 \| \| 0.913477 \| \| 1.267231 \| \| -1.25116 \| \| -1.30032 \| \| 0.369736 \| \| -0.39761 \| \| 1.704941 \| \| 1.65169 \| \| 1.273263 \| \| 2.742788 \| \| 4.170197 \| \| 2.399783 \| \| 3.001462 \| \| 5.214437 \| \| 5.864754 \| \| 5.371988 \| \| 2.723654 \| \| 0.338757 \| \| -1.00258 \| \| 0.374513 \| \| -1.02252 \| \| 1.91278 \| \| 2.186703 \| \| 1.318538 \| \| 1.274373 \| \| -1.24722 \| \| -2.16604 \| \| -2.07521 \| \| 0.64454 \| \| -0.73447 \| \| -0.71922 \| \| 2.627231 \| \| 0.93785 \| \| 0.310941 \| \| 4.321473 \| \| 4.385601 \| \| 1.322288 \| \| 2.806207 \| \| 2.440292 \| \| 4.693203 \| | \| 0 \| \| --- \| \| 1.537418 \| \| 1.862189 \| \| 0.790601 \| \| -0.36801 \| \| 2.091051 \| \| 2.099767 \| \| 1.675553 \| \| 0.473638 \| \| -0.56209 \| \| -1.47128 \| \| -2.54769 \| \| -3.78109 \| \| -1.67753 \| \| -1.75838 \| \| -2.87747 \| \| -4.0757 \| \| -1.82999 \| \| -0.867 \| \| -3.06556 \| \| -0.80483 \| \| -0.38034 \| \| -0.39626 \| \| 2.872229 \| \| 1.815445 \| \| 1.188797 \| \| 1.746783 \| \| 1.78206 \| \| 3.18615 \| \| 3.195387 \| \| 1.752382 \| \| 2.102968 \| \| 1.197187 \| \| -1.31094 \| \| -0.67351 \| \| -2.67876 \| \| -2.3509 \| \| -3.71078 \| \| -2.61168 \| \| -0.86597 \| \| -3.03314 \| \| -2.71428 \| \| -4.32438 \| \| -3.71221 \| |

Conformer **2-10**

| Atom | X | Y | Z |
| --- | --- | --- | --- |
| \| C \| \| --- \| \| C \| \| C \| \| C \| \| C \| \| C \| \| C \| \| O \| \| C \| \| C \| \| C \| \| C \| \| O \| \| C \| \| C \| \| C \| \| O \| \| C \| \| O \| \| O \| \| H \| \| H \| \| H \| \| H \| \| H \| \| H \| \| H \| \| H \| \| H \| \| H \| \| H \| \| H \| \| H \| \| H \| \| H \| \| H \| \| H \| \| H \| \| H \| \| H \| \| H \| \| H \| \| H \| \| H \| | \| 0 \| \| --- \| \| 0 \| \| 1.500363 \| \| 2.137915 \| \| 1.167979 \| \| -0.67974 \| \| -0.70146 \| \| -0.60625 \| \| 3.518987 \| \| 4.650326 \| \| 1.259012 \| \| 0.228315 \| \| 0.879443 \| \| 2.391872 \| \| 1.855802 \| \| 3.492055 \| \| 3.238881 \| \| 2.939899 \| \| 3.116181 \| \| 3.700076 \| \| 2.871516 \| \| -0.96008 \| \| 0.183467 \| \| 1.71229 \| \| 1.904389 \| \| 2.168078 \| \| -0.24676 \| \| -1.7474 \| \| -0.58668 \| \| -0.24553 \| \| -1.76249 \| \| -1.05735 \| \| 3.588387 \| \| 4.639088 \| \| 5.617572 \| \| -0.38922 \| \| -0.43707 \| \| 0.175747 \| \| 1.349989 \| \| 1.101834 \| \| 3.589872 \| \| 4.462675 \| \| 2.440124 \| \| 3.513395 \| | \| 0 \| \| --- \| \| 0 \| \| 0 \| \| 0.927516 \| \| 0.893451 \| \| 1.266971 \| \| -1.25174 \| \| -1.29481 \| \| 0.434041 \| \| 1.094038 \| \| 1.597765 \| \| 1.445366 \| \| 1.226253 \| \| 2.617615 \| \| 3.966825 \| \| 2.027907 \| \| 2.349524 \| \| 5.008958 \| \| 5.894855 \| \| 4.88643 \| \| 2.830866 \| \| 0.359706 \| \| -1.00598 \| \| 0.34937 \| \| -1.01902 \| \| 1.947588 \| \| 2.187072 \| \| 1.27392 \| \| 1.318046 \| \| -2.16554 \| \| -1.2547 \| \| -2.10454 \| \| -0.52873 \| \| 2.053254 \| \| 0.681619 \| \| 2.346 \| \| 0.594069 \| \| 0.966676 \| \| 3.894986 \| \| 4.356198 \| \| 0.941846 \| \| 2.466075 \| \| 1.811479 \| \| 4.056886 \| | \| 0 \| \| --- \| \| 1.53647 \| \| 1.855216 \| \| 0.804728 \| \| -0.37766 \| \| 2.097836 \| \| 2.084127 \| \| 3.500901 \| \| 0.456738 \| \| 0.740622 \| \| -1.53605 \| \| -2.6385 \| \| -3.88583 \| \| -1.77933 \| \| -2.28763 \| \| -2.6864 \| \| -4.05858 \| \| -2.40352 \| \| -1.57923 \| \| -3.5135 \| \| -0.81815 \| \| -0.38347 \| \| -0.39519 \| \| 2.872868 \| \| 1.778245 \| \| 1.210193 \| \| 1.691812 \| \| 1.852436 \| \| 3.18748 \| \| 1.686524 \| \| 1.808332 \| \| 3.797109 \| \| -0.0464 \| \| 1.248822 \| \| 0.47089 \| \| -2.71619 \| \| -2.47746 \| \| -4.50883 \| \| -3.2574 \| \| -1.59099 \| \| -2.58835 \| \| -2.42972 \| \| -4.27903 \| \| -4.02187 \| |
